# Supplementary material for: Sequential induction of auxin efflux and influx carriers regulates lateral root emergence
Source: Mol Syst Biol. 2013 Oct 22;9:699. doi: 10.1038/msb.2013.43 (PMC3817398; doi:10.1038/msb.2013.43)
Supplement: Supplementary Information [file msb201343-s1.pdf]

Sequential induction of auxin efflux and influx carriers regulates lateral root emergence

Supporting Modelling Information

Benjamin Péret[1,2,3,$], Alistair M. Middleton[1,2,4,11,$], Andrew P French[1,2], Antoine Larrieu[1,2], Anthony Bishopp[1,2,5], Maria Njo[6], Darren M Wells[1,2], Silvana Porco[1,2], Nathan Mellor[1,2], Leah R Band[1,2,4], Ilda Casimiro[7], Jürgen Kleine-Vehn[6], Steffen Vanneste[6], Ilkka Sairanen[8], Romain Mallet[1,2], Göran Sandberg[8], Karin Ljung[9], Tom Beeckman[6], Eva Benkova[6], Jiří Friml[6], Eric Kramer[10], John R King[1,4], Ive De Smet[2], Tony Pridmore[1], Markus Owen[1,4] and Malcolm J Bennett[1,2,*]

1 Centre for Plant Integrative Biology, University of Nottingham, LE12 5RD, UK

2 Division of Plant and Crop Sciences, School of Biosciences, University of Nottingham, LE12 5RD, United Kingdom

3 Unité Mixte de Recherche 7265, Commissariat à lEnergie Atomique et aux Energies Alternatives, Centre National de la Recherche Scientifique, Aix-Marseille Université, Laboratoire de Biologie du Dveloppement des Plantes, 13108 Saint-Paul-lez-Durance, France

4 Centre for Mathematical Medicine and Biology, School of Mathematical Sciences, University of Nottingham, Nottingham, NG7 2RD, UK

5 Institute of Biotechnology and Department of Biosciences, University of Helsinki, FIN-00014 Helsinki, Finland

6 Department of Plant Systems Biology, Flanders Institute for Biotechnology, B-9052 Ghent, Belgium - Department of Plant Biotechnology and Genetics, Ghent University, B-9052 Ghent, Belgium

7 Universidad de Extremadura, Facultad de Ciencias, Badajoz, Spain

8 UmeåPlant Science Centre, Department of Forest Genetics and Plant Physiology, Swedish University of Agricultural Sciences, SE-901 83 Umeå, Sweden

9 UmeåPlant Science Centre, Department of Plant Physiology, UmeåUniversity, SE-901 87 Umeå, Sweden

10 Simons Rock College, Physics Department, Simon's Rock College, Great Barrington, MA 01201, USA

11 Present address: University of Heidelberg, Im Neuenheimer Feld 267, 69120 Heidelberg.

$ These authors contributed equally to this work

***** Corresponding author

Contents

1 Experimental observations **5**

1.1 Auxin response and gene regulation . . . 5

1.2 Auxin transport . . . 5

2 Mathematical modelling **5**

2.1 Gene network models . . . 6

2.1.1 Model version one: *LAX3* is a primary response gene and there is no *PIN3/AEC* . . 6

2.1.2 Model version two: *LAX3* and *PIN3/AEC* are both primary response genes . . . 9

2.1.3 Model version three: *LAX3* is secondary response and *PIN3* is primary response . . 9

2.2 Modelling auxin transport in realistic tissue geometries . . . 10

2.2.1 Extracting the model geometry . . . 11

2.2.2 Modelling auxin transport . . . 11

2.2.3 Boundary conditions . . . 14

3 Parameter Values **14**

4 Numerical simulations of the model **15**

4.1 Wild-type plants . . . 15

4.2 Cell division . . . 15

4.3 Auxin dose response experiments . . . 15

4.4 Steady-states for various LRP auxin supply rates (ω_{auxin}) . . . 16

4.5 Simulation of NOA and NPA treatments . . . 16

5 Analytical treatment of the steady-state problem **17**

5.1 Auxin supplied by the LRP . . . 18

5.2 Exogenous auxin treatment . . . 19

5.3 Conditions on the sharpness of the LAX3 steady-state response function . . . 19

6 Hill function fits **20**

7 Supplementary Modelling Figures **22**

7.1 Additional results and information (Figures M1-M6) . . . 22

7.2 Steady-state plots (Figures M7-M25) . . . 27

7.3 Simulations with no sigmoidal response . . . 27

7.4 Simulations for model version one (no PIN expression) . . . 28

7.4.1 One auxin source file . . . 28

7.4.2 Three auxin source files . . . 31

7.5 Simulatons for model version two and three (with PIN1 expression) . . . 34

7.5.1 One source file . . . 34

7.5.2 Three source files . . . 37

7.6 Simulatons for model version three (LAX3 and PIN3 function blocked) . . . 40

7.6.1 One source file 40
7.6.2 Three source files 43

Development of the model

1 Experimental observations

1.1 Auxin response and gene regulation

Auxin functions in part by binding to its receptor TIR1 and mediating the degradation of Aux/IAA repressor proteins, of which 29 family members have been identified in *Arabidopsis thaliana* [6, 28]. Most, but not all, auxin responsive genes have AuxRE (auxin response element) binding sites located in their promoter region [12]. ARF (auxin response factor) oligomers can bind to AuxREs and either activate or inhibit the auxin responsive genes [29, 12], whereas Aux/IAAs can bind ARFs and antagonise ARF-mediated gene regulation. Degradation of Aux/IAA proteins can normally occur within minutes (see [22] and references therein). Genes that are direct targets of the ARFs and Aux/IAAs can in turn respond to auxin on the timescale of tens of minutes, and as such are typically referred to as primary response genes. These includes genes encoding the Aux/IAAs themselves, this constituting a negative feedback loop. Middleton *et al.* [22] developed the first model of the Aux/IAA negative feedback loop and its response to changes in auxin. Some primary response genes encode proteins (e.g. transcription factors) that regulate so-called secondary response genes. One way to test experimentally whether a gene is primary or secondary responsive is to block mRNA translation chemically, using cycloheximide (CHX). If the gene is a primary response one, then it should be upregulated by CHX treatment. This is because synthesis of Aux/IAA proteins is blocked, and so their levels decrease (due to auxin-mediated degradation), thus freeing (activating) ARF proteins to up-regulate primary response genes. However, secondary response genes will not be upregulated (because the synthesis of the required intermediate is prevented by the CHX treatment).

1.2 Auxin transport

Auxin is a weak acid and can take either a protonated form IAA^- or an anionic form IAAH [18]. The former can pass through cell membranes by diffusion, whereas the latter requires specific influx and efflux transporters for movement across membranes. The ratio of anionic to protonated auxin depends on the pH: in the apoplast (which is acidic), auxin exists in both protonated and anionic forms, whereas in the cytoplasm (which is basic) auxin is largely anionic. Thus, auxin can move from the apoplast into the cytoplasm either passively or via influx carriers, but requires an efflux tranporter for it to leave the cell at a comparible rate (i.e. this being 'acid trapping'). Key influx and efflux carriers are members of the AUX1/LAX and PIN gene families, respectively. Depending on the particular developmental context, PIN family members can be localised to a specific cell membrane (i.e. be polarly localised) [15].

2 Mathematical modelling

We underwent several iterations of the model-experiment cycle, and its by doing so developed three different model versions, these being summarised in Table M1. We first discuss the regulatory network models before describing our model for auxin transport (which is common to all model variants). Thus, in Section 2.1.1

we describe the first version of the model, which does not include the auxin efflux carrier PIN3. In Section 2.1.2 we describe model version two, which is where we first introduce *PIN3* as a component of the model. It is worth stressing that, prior to characterizing *PIN3* experimentally, we hypothesised (in model version two) the existence of such a network component by introducing a hypothetical auxin efflux carrier which we denote *AEC*. Hence, for model version two, we refer to this entity as *PIN3/AEC*. However, in the governing equations, for the sake of brevity, we simply write *PIN3*. Then, in Section 2.1.3 we describe the gene network model adopted in the final version of the model (whereby *PIN3* is modelled as a primary response gene and *LAX3* as a secondary response one). In Section 2.2 we discuss our model of auxin transport (this being for all model versions). An illustration of the spatial localisation of the various transport components is provided in Figure M1 (note all figures and tables appearing in this document have prefix M). The various simulations performed with these models (including the simulations of the LAX3 dose response and the simulation of the NOA treatments) are described in Section 4. Unless otherwise stated, default parameter values are provided in Table M2. In each case, the governing equations were solved using `ode15s`, a MATLAB subroutine for solving stiff problems. Solutions were then visualised using MEDIT [8] (see main text).

2.1 Gene network models

2.1.1 Model version one: *LAX3* is a primary response gene and there is no *PIN3/AEC*

In model version one we assumed that *LAX3* encoded a primary response gene (we later find it to be a secondary response gene and include this in model version three) and that there is no *PIN3/AEC* in the model (this is included in model versions two and three). The interactions captured by the model are illustrated in main text Figure 4A. In the context of lateral root emergence, the relevant Aux/IAA and ARF family members are IAA14 and ARF7 [26]. In the model, we write $[\mathrm{Y}_m]_i$ for the concentration of an mRNA encoded by gene Y in cell i, and $[\mathrm{Y}]_i$ for the concentration of the corresponding protein (noting that we extend this notation to denote the concentration of the hormone auxin). Thus, we assume that each gene Y in the network (namely *IAA14* and *LAX3*) is a direct target of ARF7 and IAA14. Thus, the rate of transcription of gene Y in cell i is an increasing function of $[\mathrm{ARF7}]_i$ and a decreasing one of $[\mathrm{IAA14}]_i$. In this way, IAA14 antagonises (and therefore has an inhibitory effect on) ARF7 mediated activation of gene Y. This is activation is counter-acted by degradation of the mRNA, which occurs at rate μ_Y; mRNA levels are normalised such that their maximal steady-state concentration is equal to one:

$$\frac{d[\mathrm{Y_m}]_i}{dt} = \mu_\mathrm{Y}\left(\frac{([\mathrm{ARF7}]/\theta_\mathrm{Y})^m}{1+([\mathrm{ARF7}]/\theta_\mathrm{Y})^m+\sum_{n=1}^{m}([\mathrm{ARF7}][\mathrm{IAA14}]/\psi_{\mathrm{Y}_n})^n} - [\mathrm{Y_m}]_i\right), \quad \text{for Y=IAA14, LAX3;} \tag{2.1}$$

θ_Y is the ARF–DNA promoter dissociation constant; ψ_{Y_n} is the IAA14–ARF7-DNA dissociation constant (when there are n ARF7-IAA14 dimers bound to the promoter). A default assumption, in the absence of more detailed information, is that induction of gene expression is not co-operative (i.e. so $m = 1$). We maintain this assumption for *IAA14* throughout. However, dose response experiments with auxin (see main text Figure 4H) indicate that there is a sigmoidal relationship between LAX3 and auxin (see also Section 5). This can be captured in the model by choosing m in (2.1) to be larger than unity; see Figure M3.

Model version	Gene network model equations and variables	Section	Description and Notes
Version 1	mRNA concentrations: [$LAX3_m$] (2.5b), [$IAA14_m$] (2.5a), and associated proteins: [LAX3] and [IAA14] (2.6, 2.7)	2.1.1	*LAX3* is a primary response gene (and there is no PIN3). A diagram of the gene network is provided in main text Figure 4A. Default parameters given in Table M2. Parameters in (2.5b) are calculated using (2.15). In the main text, we discuss two different versions of this model, one where the Hill Coefficient in (2.5b) $m = 1$ (see main), and one where $m = 3$ (see main text Figures 4G and 4I). Steady state solutions are plotted in Supplementary Modelling Figures M8-M13.
Version 2	mRNA concentrations: [$IAA14_m$] (2.5a), [$LAX3_m$] (2.5b), [$PIN3_m$] (2.8), and associated proteins: [IAA14], [PIN3] and [LAX3] (2.6, 2.7 and 2.9)	2.1.2	Both *PIN3/AEC* and *LAX3* are primary response genes. A diagram of the gene network is provided in main text Figure 4E. Default parameters given in Table M2. Parameters in (2.5b) are calculated using (2.15). By doing so, model version 2 and model version 3 have the same steady state solutions. Solutions to this model are provided in Figure 5D,E (although in Figure 5D PIN3 is only polarized towards neighbouring cortical cells). Steady state solutions are plotted in Supplementary Modelling Figures M14-M25
Version 3	mRNA concentration: [$IAA14_m$] (2.5a),[$PIN3_m$] (2.8), [X_m] (2.10), [$LAX3_m$] (2.12), and associated protein concentrations [IAA14], [X], [LAX3], [PIN3] (2.6, 2.7 and 2.9)	2.1.3	*PIN3* is a primary response gene and *LAX3* is a secondary response one. Interactions are illustrated in Figure 7. Default parameters given in Table M2. Steady state solutions are plotted in Supplementary Modelling Figures M14-M25.

Table M1: Summary of the various model version discussed in the paper, and how they relate to the main text figures. Equations for the (cortex) gene network are provided. These are coupled to a model of auxin transport (namely (2.21)-(2.24)), discussed in Section 2.2. We note that, in the case of model version 1, where there is no *PIN3/AEC*, we can simply set $[\text{PIN3}]_i \equiv 0$ in (2.21)-(2.24) to obtain the appropriate transport equations.

We further assume that all mRNAs are translated into proteins at a rate that is proportional to their concentration (with constant of proportionality δ_{Y}), and for all proteins (except IAA14, which is targeted for degradation by auxin, see below). Similarly, we take degradation to occur at a rate that is proportional to the concentration of the protein (with proportionality constant γ_{Y}). We will apply these assumptions to other network components that appear in other model versions (namely LAX3 in this version of the model, PIN3 in model version two and three and factor X in model version three), and so we write the governing equations in terms of Y. However, in model version one, the only relevant network component is LAX3. Thus, $[\mathrm{Y}]_i$ is governed by:

$$\frac{d[\mathrm{Y}]_i}{dt} = \delta_{\mathrm{Y}}[\mathrm{Y_m}]_i - \gamma_{\mathrm{Y}}[\mathrm{Y}]_i, \quad \text{for Y=LAX3.} \tag{2.2}$$

To capture the auxin-mediated degradation of IAA14 proteins, we use a simplified version of the model developed in Middleton *et al.* [22], namely we take the protein decay rate to be a saturable (monotonically increasing) function of auxin and IAA14. Thus $[\mathrm{IAA14}]_i$ is governed by:

$$\frac{d[\mathrm{IAA14}]_i}{dt} = \delta_{\mathrm{IAA14}}[\mathrm{IAA14_m}]_i - l_m\frac{[\mathrm{auxin}]_i[\mathrm{IAA14}]_i}{1+K[\mathrm{auxin}]_i[\mathrm{IAA14}]_i}. \tag{2.3}$$

where the maximal rate of decay is l_m and decay is half-maximal at $[\mathrm{auxin}][\mathrm{IAA14}] = 1/K$.

Since the gene *ARF7* is not regulated by auxin, we do not include transcriptional regulation of ARF7 in the model. We rather assume that that the proteins are synthesised and degraded at constant rates δ_{ARF7} and γ_{ARF7} respectively:

$$\frac{d[\mathrm{ARF7}]_i}{dt} = \delta_{\mathrm{ARF7}} - \gamma_{\mathrm{ARF7}}[\mathrm{ARF7}]_i. \tag{2.4}$$

Although ARF7 may bind IAA14 when it is not bound to a promoter [11, 1], we assume that this occurs on much faster timescales than those of protein synthesis and degradation. Thus, these binding events should have negligible impact on ARF7 levels. We take the degradation of the various proteins, and translation of the mRNAs, to be rapid when compared to the rate of mRNA transcription and degradation. Thus, we set the left-hand sides of (2.2), (2.3) and (2.4) to zero (this being the quasi steady-state assumption). Furthermore, we assume that auxin levels are not saturating in (2.3), i.e. that $[\mathrm{auxin}][\mathrm{IAA14}] \ll 1/K$. It follows from these considerations that the equations governing the LAX3 gene network (for model version one) in cortical cell i simplify to

$$\frac{d[\mathrm{IAA14_m}]_i}{dt} = \mu_{\mathrm{IAA14}}\left(\frac{[\mathrm{ARF7}]_i/\theta_{\mathrm{IAA14}}}{1+[\mathrm{ARF7}]_i/\theta_{\mathrm{IAA14}}+[\mathrm{IAA14}]_i[\mathrm{ARF7}]/\psi_{\mathrm{IAA14}}} - [\mathrm{IAA14_m}]\right), \tag{2.5a}$$

$$\frac{d[\mathrm{LAX3_m}]_i}{dt} = \mu_{\mathrm{LAX3}}\left(\frac{([\mathrm{ARF7}]/\theta^*_{\mathrm{LAX3}})^m}{1+([\mathrm{ARF7}]/\theta^*_{\mathrm{LAX3}})^m+\sum_{n=1}^m([\mathrm{ARF7}][\mathrm{IAA14}]/\psi^*_{\mathrm{LAX3}_n})^n} - [\mathrm{LAX3_m}]_i\right), \tag{2.5b}$$

$$[\mathrm{LAX3}]_i = \phi_{\mathrm{LAX3}}[\mathrm{LAX3_m}]_i, \quad \text{where} \quad \phi_{\mathrm{LAX3}} = \frac{\delta_{\mathrm{LAX3}}}{\gamma_{\mathrm{LAX3}}}, \tag{2.6}$$

and

$$[\mathrm{IAA14}]_i = \frac{\delta_{\mathrm{IAA14}}[\mathrm{IAA_m}]_i}{l_m[\mathrm{auxin}]_i}. \tag{2.7}$$

In (2.5b) we have appended stars to the θ and ψ parameters, so as to distinguish these from the parameters that appear in model version three (see below). Furthermore, we treat $[\text{ARF7}]_i = [\text{ARF7}]$ ($= \delta_{\text{ARF7}}/\gamma_{\text{ARF7}}$) as a constant in the model (i.e. so that the concentration of ARF7 is spatially uniform). Equations (2.5)-(2.6) couple to equations governing the transport of auxin (namely (2.21)-(2.24), see Section 2.2). We proceed by solving (2.5)-(2.6) (ensuring that (2.15) is satisfied, see later) for the gene network (in a cortical cell) and (2.21)-(2.24) for the transport of auxin (setting $[\text{PIN3}]_i \equiv 0$ or equivilently $P_{\text{PIN3}} = 0$ in the governing equations; see Section 2.2). The spatial localisation of the various influx and efflux carriers in this version of the model is illustrated in Figure M1a.

2.1.2 Model version two: *LAX3* and *PIN3/AEC* are both primary response genes

Here, as with model version one, *LAX3*, is modelled as a primary response gene (instead of a secondary response one, this being the case in the third and final version of the model, see Section 2.1.3). However, unlike model version one, we here introduce *AEC/PIN3* into the model (as a primary response gene). The interactions captured by the model are illustrated in the main text Figure 4A. We stress that, for the sake of simplicity, we refer to AEC/PIN3 as just PIN3 in the various governing equations. It follows from Section 2.1.1 that the equations governing *PIN3* mRNA ($[\text{PIN3}_\text{m}]_i$) and PIN3 protein ($[\text{PIN3}]_i$) in cell i are

$$\frac{d[\text{PIN3}_\text{m}]_i}{dt} = \mu_{\text{PIN3}} \left(\frac{[\text{ARF7}]_i/\theta_{\text{PIN3}}}{1 + [\text{ARF7}]_i/\theta_{\text{PIN3}} + [\text{IAA14}]_i[\text{ARF7}]_i/\psi_{\text{PIN}}} - [\text{PIN3}_\text{m}]_i \right), \tag{2.8}$$

and

$$[\text{PIN3}]_i = \phi_{\text{PIN3}}[\text{PIN3}_\text{m}]_i, \quad \text{where} \quad \phi_{\text{PIN3}} = \frac{\delta_{\text{PIN3}}}{\gamma_{\text{PIN3}}}, \tag{2.9}$$

In summary, the second version of the model is governed by equations (2.5)-(2.6) and (2.8)-(2.9) for the gene network (in a cortical cell) and equations (2.21)-(2.24) for the transport of auxin (these being described in Section 2.2). The spatial localisation of the various influx and efflux carrier in this version of the model is illustrated in Figure M1b (these being the same in model version 3, see below).

2.1.3 Model version three: *LAX3* is secondary response and *PIN3* is primary response

In this final version of the model, we modify model version two (see Section 2.1.2) by taking into account that *LAX3* is a secondary response gene (see main text) and is presumably activated by some unknown intermediate (which we denote X). The interactions in this model are illustrated in main text Figure 7. In the main text, we find that PIN3 is indeed a primary response gene, consistent with our (model version two) assumptions (see main text). Factor X is assumed to be encoded by a auxin primary response gene (see 2.1). The equations governing its mRNA, $[\text{X}_\text{m}]$, and protein, $[\text{X}]$, are then

$$\frac{d[\text{X}_\text{m}]_i}{dt} = \mu_X \left(\frac{[\text{ARF7}]_i/\theta_X}{1 + [\text{ARF7}]_i/\theta_X + [\text{IAA14}]_i[\text{ARF7}]_i/\psi_X} - [\text{X}_\text{m}]_i \right), \tag{2.10}$$

and

$$[\text{X}]_i = \phi_\text{X}[\text{X}_\text{m}]_i, \quad \text{where} \quad \phi_\text{X} = \frac{\delta_\text{X}}{\gamma_\text{X}}. \tag{2.11}$$

To model *LAX3*, we use a Hill function (with Hill coefficient m) to capture how its rate of transcription depends on the concentration of factor X protein. Thus, the concentration of *LAX3* mRNA in cell i is governed by:

$$\frac{d[\mathrm{LAX3_m}]_i}{dt} = \mu_{\mathrm{LAX3}} \left(\frac{([\mathrm{X}]_i/\theta_{\mathrm{LAX3}})^m}{1 + ([\mathrm{X}]_i/\theta_{\mathrm{LAX3}})^m} - [\mathrm{LAX3_m}]_i \right). \tag{2.12}$$

The equation governing the protein concentration of LAX3 is given by (2.6). Thus, model version three comprises (2.5a), (2.8), (2.10) and (2.12) for the various mRNAs in the gene network model and (2.6), (2.7), (2.9), (2.11) for the associated proteins. These are coupled to equations (2.21)-(2.24), which govern the transport of auxin (these being described in Section 2.2). The spatial localisation of the various influx and efflux carriers in this version of the model is illustrated in Figure M1b (these being the same in model version 2, see above).

We note that the raison d'etre for including the intermediate gene (transcription factor X) in the final version of the regulatory network model is to cause the auxin-mediated induction of *LAX3* to be delayed (but not alter its steady-state levels). To understand this, suppose we were to assume that X is upregulated instantly. In this case there would be no such delay and LAX3 would in effect act as a primary response gene. This can be seen by taking the limit $\mu_X \to \infty$ in (2.10), to obtain

$$X_m = \frac{[\mathrm{ARF7}]/\theta_X}{1 + [\mathrm{ARF7}]/\theta_X + [\mathrm{IAA14}][\mathrm{ARF7}]/\psi_X}. \tag{2.13}$$

Adopting (2.13) is equivalent to assuming that the level of transcription factor X is quasi-steady (i.e. that it instantly attains its steady state level for a given concentration of IAA14). Upon substituting (2.13) into (2.12) and recalling that $X = \phi_X X_m$, we find that

$$\frac{d[\mathrm{LAX3_m}]}{dt} = \mu_{\mathrm{LAX3}} \left(\frac{([\mathrm{ARF7}]\phi_X/(\theta_X\theta_{\mathrm{LAX3}}))^m}{([\mathrm{ARF7}]\phi_X/(\theta_X\theta_{\mathrm{LAX3}}))^m + (1 + [\mathrm{ARF7}]/\theta_X + [\mathrm{IAA14}][\mathrm{ARF7}]/\psi_X)^m} - [\mathrm{LAX3_m}] \right). \tag{2.14}$$

This equation is of the same form as (2.5b). Moreover if write the parameters in (2.5b) in terms of the ones in (2.14), namely

$$\theta^*_{\mathrm{LAX3}} = \frac{\theta_{\mathrm{LAX3}}(\theta_X + [\mathrm{ARF7}])}{\phi_X}, \qquad \psi^*_{\mathrm{LAX3}_n} = \psi_X \left(1 + \frac{[\mathrm{ARF7}]}{\theta_X}\right) \binom{m}{n}^{-1/n}, \tag{2.15}$$

then upon substituting (2.15) into (2.5b) we obtain (2.14). Thus, we choose the parameters θ^*_{LAX3} and $\psi^*_{\mathrm{LAX3}_n}$ in (2.5b) according to (2.15) thus ensuring that the steady states of this (third) version of the model will match exactly those of the second version (i.e. where *LAX3* is treated as a primary response gene). This allows us easily to understand what the precise affect of treating *LAX3* as either a primary or a secondary response gene is (see main text).

2.2 Modelling auxin transport in realistic tissue geometries

We model the transport of auxin in realistic three-dimensional tissue geometries that were extrapolated from confocal images (see Section 2.2.1 for details). This allows us to capture natural variations in cell volume, surface area and connectivity (i.e. neighbourhood relations between individual cells). The geometries used

in other models of auxin transport range from relatively simples ones, where cells are treated as boxes that are arranged on a regular n-dimensional grid (for n=1,2 or 3) through to two or three dimensional vertex-based representations of cells (see for example [13, 10, 19]) and [5, 14, 16, 24] for recent reviews). Our model for auxin transport is an extension of the one developed by Goldsmith [9] and Kramer [17, 27]. Analogous two-dimensional vertex-based approaches have also been been presented in [23] and [25].

2.2.1 Extracting the model geometry

The root is composed of several concentric layers (the key ones here being the pericycle, endodermis, cortex and epidermis, main text Figure 1A), and each layer is organised into several cell files that extend along the length of the root. Cross-sectional images of plant roots were obtained using light microscopy (Figure 4B). These were then manually segmented (Figure 4C), and then extruded to recreate realistically the longitudinal axis (i.e. such that cell files are staggered, Figure 1B). A triangular mesh of the tissue was generated using the CGAL library [2]. Here, the mesh consists of polygonal surfaces representing the interfaces between cells; whilst the apoplast (the region between cells' plasma membranes) is not included as a separate compartment, we do capture its effect in the governing equations (see (2.20) and (2.23) below). We assume that the concentration of auxin within each cell is uniform. Each cell i has several neighbouring cells j. Since the geometry is static, the relevant properties of the mesh, such as the volume of each cell i (V_i), the surface area shared between two cells i and j ($S_{i,j}$) or the neighbour relations between cells (i.e. the set of cells j that neighbour cell i, which we denote $\mathcal{N}_i$) are calculated prior to simulation of the model. These variables then appear in the equations governing auxin transport – namely (2.21)-(2.24).

2.2.2 Modelling auxin transport

Illustrated in Figure M1 is the spatial localization (and polarity) of the various auxin transport components (namely, influx and efflux carriers) that appear in the different model versions. We note that in this work we hypothesize the existence of an auxin-inducible auxin-efflux carrier in the cortex, which we denote AEC; we subsequently identified it to be PIN3. For the sake of brevity, we denote PIN3/AEC as simply PIN3 in the governing equations (2.21)-(2.24) given below. We note that model version one (unlike versions two and three) does not contain PIN3/AEC. However, in this case, we simply set $[\mathrm{PIN}]_i \equiv 0$ in (2.21)-(2.24). In this work (see main text), we find that the auxin regulating cortical *LAX3* and *PIN3/AEC* are largely supplied by just one of the three pericycle cells that will later form the lateral root primordium (LRP; namely, the centre one – see Figure M1). LRP cells express the influx carrier AUX1 [21, 19]. However, for the auxin provided by such a source to enter the cortex, it must pass through the endodermis. Passive diffusion alone is not enough to account for this. We therefore adopt the biologically plausible assumption that it is transported through the endodermis into the cortex by an unidentified auxin efflux carrier (which we denote UEC). In the model, AUX1 and UEC are not regulated by auxin (unlike PIN3/AEC and LAX3). To capture the fact that the expression of each carrier species can be restricted to a particular cell type or cell membrane, we introduce the parameters $\Phi^{\chi}_{k,l}$, where χ is either PIN3/AEC, LAX3, AUX1 or UEC, and k and l are the indices of two cells. If the cells neighbour each other, let the (ordered) pair (l, k) denote the membrane region of cell l that faces cell k. We let $\Phi^{\chi}_{l,k} = 1$ if carriers of type χ can localize to membrane (l, k). We set $\Phi^{\chi}_{l,k} = 0$ either if cells l and k are neighbours but species χ does not localise

to that membrane, or if the two cells are not neighbours (and hence membrane (l,k) does not exist). We note that $\Phi^{\chi}_{l,k}$ does not necessarily equal $\Phi^{\chi}_{k,l}$.

Here, we follow [17] in modelling the flux of auxin across a cell membrane, from a given cell, k, into an adjacent apoplast compartment (that lies, say, between cell k and cell l). The passive (diffusion) flux, J_{IAAh}, is driven by the difference in the concentrations of protonated auxin. Denoting the proportions of protonated auxin in the cytoplasm and apoplast by A_1 and B_1 respectively, the passive flux is given by

$$J_{\mathrm{IAAh}_{k,l}} = P_{\mathrm{IAAh}}(B_1[\mathrm{auxin}]_k - A_1[\mathrm{auxin}]^{\mathrm{apo}}_{k,l}), \tag{2.16}$$

where $[\mathrm{auxin}]_k$ is the concentration of (intracellular) auxin in cell k, and $[\mathrm{auxin}]^{\mathrm{apo}}_{l,k}$ is the concentration in the apoplast compartment between cells l and k. We not that $J_{\mathrm{IAAh}_{k,l}}$ does not necessarily equal $J_{\mathrm{IAAh}_{l,k}}$. The proportion of protonated auxin depends on the difference between the pH and auxin's dissociation constant, pK, such that the values of A_1 and B_1 are given by

$$A_1 = \frac{1}{1+10^{\mathrm{pH}^{\mathrm{apo}}-\mathrm{pK}}}, \qquad B_1 = \frac{1}{1+10^{\mathrm{pH}^{\mathrm{cyt}}-\mathrm{pK}}}, \tag{2.17}$$

where $\mathrm{pH}^{\mathrm{apo}}$ denotes the apoplastic pH and $\mathrm{pH}^{\mathrm{cyt}}$ the cytoplasmic one.

Carrier mediated auxin fluxes are typically modelled using the Goldman-Hodgkin-Katz equations, which incorporate fluxes driven by the electrochemical gradient across the cell membrane (see, for example, [27]). These result in the LAX3/AUX1 and PIN3/AEC/UEC mediated fluxes being given, respectively, by

$$J_{\mathrm{LAX3}_{k,l}} = P_{\mathrm{LAX3}}[\mathrm{LAX3}]_k\Phi^{\mathrm{LAX3}}_{k,l}(B_2[\mathrm{auxin}]_k - A_2[\mathrm{auxin}]^{\mathrm{apo}}_{k,l}), \tag{2.18a}$$
$$J_{\mathrm{AUX1}_{k,l}} = P_{\mathrm{AUX1}}\Phi^{\mathrm{AUX1}}_{k,l}(B_2[\mathrm{auxin}]_k - A_2[\mathrm{auxin}]^{\mathrm{apo}}_{k,l}), \tag{2.18b}$$
$$J_{\mathrm{PIN3}_{k,l}} = P_{\mathrm{PIN3}}[\mathrm{PIN3}]_k\Phi^{\mathrm{PIN3}}_{k,l}(B_3[\mathrm{auxin}]_k - A_3[\mathrm{auxin}]^{\mathrm{apo}}_{k,l}), \tag{2.18c}$$
$$J_{\mathrm{UEC}_{k,l}} = P_{\mathrm{UEC}}\Phi^{\mathrm{UEC}}_{k,l}(B_3[\mathrm{auxin}]_k - A_3[\mathrm{auxin}]^{\mathrm{apo}}_{k,l}), \tag{2.18d}$$

where $P_{\mathrm{LAX3}}[\mathrm{LAX3}]$, P_{AUX1}, $P_{\mathrm{PIN3}}[\mathrm{PIN3}]$ and P_{UEC} are the effective permeabilities of the cell membrane due to LAX3, AUX1, PIN3/AEC and UEC respectively. The constants A_i, B_i for $i = 2,3$ in (2.18) are defined by

$$A_2 = \frac{q(-\phi)}{1+10^{-\mathrm{pH}^{\mathrm{apo}}+\mathrm{pK}}}, \qquad A_3 = \frac{q(\phi)}{1+10^{-\mathrm{pH}^{\mathrm{apo}}+\mathrm{pK}}}, \tag{2.19}$$
$$B_2 = \frac{q(\phi)}{1+10^{-\mathrm{pH}^{\mathrm{cyt}}+\mathrm{pK}}}, \qquad B_3 = \frac{q(-\phi)}{1+10^{-\mathrm{pH}^{\mathrm{cyt}}+\mathrm{pK}}}.$$

Here, $\phi = FV/RT$ is a dimensionless constant, where V is the membrane potential, F is the Faraday constant, R is the gas constant and T is the temperature. A derivation of these flux terms, (2.18), can be found in Appendix B of the recent paper [3]. In using (2.18), we assume that the concentrations of LAX3 and PIN3/AEC protein in the membrane are proportional to their respective intracellular concentrations. Whilst in reality, this should be a saturable relationship, we assume the physiological levels of the carrier are far below the concentration where this becomes important. The concentrations of UEC and AUX1 (and hence their associated permeabilities) are assumed to be constants. As noted above (see also Figure M1), the various influx and efflux carriers are spatially distributed according to tissue type. However, this is captured by our construction of the variables $\Phi^{\chi}_{k,l}$ (see above). This allows us to keep the equations that

follow as general as possible (so that they hold for any cell geometry). We thus sum over the relevant flux terms and obtain the equations governing the concentration of auxin in the apoplast compartment joining cells l and k ($[\text{auxin}]^{\text{apo}}_{l,k}$) in the form

$$\begin{aligned}\frac{d[\text{auxin}]^{\text{apo}}_{l,k}}{dt} =& -\frac{S_{l,k}}{V^{\text{apo}}_{l,k}}\Big(P_{\text{IAAh}}(2A_1[\text{auxin}]^{\text{apo}}_{l,k} - B_1([\text{auxin}]_l + [\text{auxin}]_k))\\ &+P_{\text{LAX3}}[\text{LAX3}]_l\Phi^{\text{LAX3}}_{l,k}(A_2[\text{auxin}]^{\text{apo}}_{l,k} - B_2[\text{auxin}]_l)\\ &+P_{\text{LAX3}}[\text{LAX3}]_k\Phi^{\text{LAX3}}_{k,l}(A_2[\text{auxin}]^{\text{apo}}_{l,k} - B_2[\text{auxin}]_k)\\ &+P_{\text{PIN3}}[\text{PIN3}]_l\Phi^{\text{PIN3}}_{l,k}(A_3[\text{auxin}]^{\text{apo}}_{l,k} - B_3[\text{auxin}]_l)\\ &+P_{\text{PIN3}}[\text{PIN3}]_k\Phi^{\text{PIN3}}_{k,l}(A_3[\text{auxin}]^{\text{apo}}_{l,k} - B_3[\text{auxin}]_k)\\ &+P_{\text{AUX1}}\Phi^{\text{AUX1}}_{l,k}(A_2[\text{auxin}]^{\text{apo}}_{l,k} - B_2[\text{auxin}]_l)\\ &+P_{\text{AUX1}}\Phi^{\text{AUX1}}_{k,l}(A_2[\text{auxin}]^{\text{apo}}_{l,k} - B_2[\text{auxin}]_k)\\ &+P_{\text{UEC}}\Phi^{\text{UEC}}_{l,k}(A_3[\text{auxin}]^{\text{apo}}_{l,k} - B_3[\text{auxin}]_l)\\ &+P_{\text{UEC}}\Phi^{\text{UEC}}_{k,l}(A_3[\text{auxin}]^{\text{apo}}_{l,k} - B_3[\text{auxin}]_k)\Big)\,,\end{aligned} \tag{2.20}$$

where $V^{\text{apo}}_{l,k}$ is the volume of the apoplast compartment connecting cells l and k. For the intracellular auxin in cell i, we write:

$$\begin{aligned}\frac{d[\text{auxin}]_i}{dt} =& V_i^{-1}\Bigg(P_{\text{IAAh}}\Bigg(A_1\sum_{j\in\mathcal{N}_j} S_{i,j}[\text{auxin}]^{\text{apo}}_{i,j} - B_1 S_i[\text{auxin}]_i\Bigg)\\ &+P_{\text{LAX3}}[\text{LAX3}]_i\Bigg(A_2\sum_{j\in\mathcal{N}_j} S_{i,j}\Phi^{\text{LAX3}}_{i,j}[\text{auxin}]^{\text{apo}}_{i,j} - B_2 S_i^{\text{LAX3}}[\text{auxin}]_i\Bigg)\\ &+P_{\text{PIN3}}[\text{PIN3}]_i\Bigg(A_3\sum_{j\in\mathcal{N}_j} S_{i,j}\Phi^{\text{PIN3}}_{i,j}[\text{auxin}]^{\text{apo}}_{i,j} - B_3 S_i^{\text{PIN3}}[\text{auxin}]_i\Bigg) \quad (2.21)\\ &+P_{\text{AUX1}}\Bigg(A_2\sum_{j\in\mathcal{N}_j} S_{i,j}\Phi^{\text{AUX1}}_{i,j}[\text{auxin}]^{\text{apo}}_{i,j} - B_2 S_i^{\text{AUX1}}[\text{auxin}]_i\Bigg)\\ &+P_{\text{UEC}}\Bigg(A_3\sum_{j\in\mathcal{N}_j} S_{i,j}\Phi^{\text{UEC}}_{i,j}[\text{auxin}]^{\text{apo}}_{i,j} - B_3 S_i^{\text{UEC}}[\text{auxin}]_i\Bigg)\Bigg)\\ &+\omega^i_{\text{auxin}} - \mu_{\text{auxin}}[\text{auxin}], \quad (2.22)\end{aligned}$$

where

$$S_i = \sum_{j\in\mathcal{N}_j} S_{i,j}, \qquad S_i^{\text{LAX3}} = \sum_{j\in\mathcal{N}_j}\Phi^{\text{LAX3}}_{i,j}S_{i,j}, \qquad S_i^{\text{PIN3}} = \sum_{j\in\mathcal{N}_j}\Phi^{\text{PIN3}}_{i,j}S_{i,j},$$
$$S_i^{\text{AUX1}} = \sum_{j\in\mathcal{N}_j}\Phi^{\text{AUX1}}_{i,j}S_{i,j}, \qquad S_i^{\text{UEC}} = \sum_{j\in\mathcal{N}_j}\Phi^{\text{UEC}}_{i,j}S_{i,j},$$

$S_{i,j}$ being the surface area of triangle j of cell i. Additionally, ω^i_{auxin} is the rate of auxin production in cell i and μ_{auxin} is the decay/deactivation rate of intracellular auxin. In the model, we assume that

all cells (except the source cell s) produce auxin at some low basal rate . Thus, for all $i \neq s$, we write $\omega^i_{\text{auxin}} = \omega^{\text{basal}}_{\text{auxin}}$. For the source cell, we write $\omega^s_{\text{auxin}} = \omega^{\text{basal}}_{\text{auxin}} + \omega_{\text{auxin}}$

Following the method presented in [23], we now simplify the auxin-transport model. Approximating the apoplast thickness to be constant, denoted λ, the volume of the apoplast can be written as $V^{\text{apo}}_{l,k} = \lambda S_{l,k}$ (where $S_{l,k}$ is the apoplast compartment's surface area). The apoplast is typically thin compared to the size of a cell, and so it is appropriate to take the limit in which $\lambda \to 0$. In this limit, the auxin concentration in each apoplast region is quasi-steady and, using (2.20), we obtain expressions for the apoplast concentrations in terms of the auxin concentrations in the neighbouring cells:

$$[\text{auxin}]^{\text{apo}}_{l,k} = \frac{(B_1 P_{\text{IAAh}} + B_2\alpha_{l,k} + B_3\beta_{l,k})[\text{auxin}]_l + (B_1 P_{\text{IAAh}} + B_2\alpha_{k,l} + B_3\beta_{k,l})[\text{auxin}]_k}{2A_1 P_{\text{IAAh}} + A_2(\alpha_{l,k} + \alpha_{k,l}) + A_3(\beta_{l,k} + \beta_{k,l})} \tag{2.23}$$

where

$$\alpha_{l,k} = P_{\text{LAX3}}[\text{LAX3}]_l \Phi^{l,k}_{\text{LAX3}} + P_{\text{AUX1}}\Phi^{l,k}_{\text{AUX1}}, \text{ and } \beta_{l,k} = P_{\text{PIN3}}[\text{PIN3}]_l \Phi^{l,k}_{\text{PIN3}} + P_{\text{UEC}}\Phi^{l,k}_{\text{UEC}}.$$

Using this approximation, the equations governing auxin transport reduce to a system of ordinary differential equations for the auxin concentration in each cell (2.21) coupled to expressions for the concentrations in the apoplast regions, (2.23).

2.2.3 Boundary conditions

For most model simulations (except when simulating the LAX3 dose response experiments, see Section 4.3), we adopt zero-flux boundary conditions, so that for all externally facing apoplast compartments we replace (2.23) with

$$[\text{auxin}]^{\text{apo}}_{l,0} = \frac{(B_1 P_{\text{IAAh}} + B_2\alpha_{l,0} + B_3\beta_{l,0})[\text{auxin}]_l}{A_1 P_{\text{IAAh}} + A_2\alpha_{l,0} + A_3\beta_{l,0}}, \tag{2.24}$$

where the index 0 denotes the root's exterior.

3 Parameter Values

A summary of the various parameters in the model (with the exception of mesh-related parameters, such as surface areas $S_{i,j}$ and cell volumes V_i), together with their default values and references (where applicable) is provided in Table M2. Estimates for many of the parameters associated with auxin transport (including the various membrane permeabilities P_{IAAh}, P_{PIN3}, P_{LAX3} and P_{AUX1},) are available and a detailed discussion of these can be found in [27]. Lateral root emergence occurs when cells have full elongated and matured. In this region, experimental data indicates that the pH of the apoplast is approximately 6 [7]; we use this value in our calculation of the constants A_i and B_i for $i = 1, 2, 3$ (see (2.17) and (2.19)). Estimates for the rate of auxin decay/deactivation (μ_{auxin}) in this context are not available; however, it is likely that this occurs on the timescale of minutes [20] and we choose μ_{auxin} accordingly. Estimates for many of the parameters associated with the gene network models are also not available. However, the simulations presented in this paper are representative of a broad range of parameter values: the only parameter for which the model was particularly sensitive to variations in m, the Hill coefficient in (2.10) and (2.5b) (see Section 4.3). In

particular, the parameters μ_{LAX3}, μ_{IAA14}, μ_{X} and μ_{PIN3} control the timescales over which changes in gene expression occurs for *LAX3*, *IAA14*, *X*, and *PIN3* respectively. These parameters were chosen to reflect that fact that such changes should occur on the order of tens of minutes to hours.

4 Numerical simulations of the model

Here we discuss the various simulations of the different model versions discussed in the main text. In particular, here we describe the initial conditions adopted in each scenario, together with details of the relevant parameter choices (see Table M2 for their default values).

4.1 Wild-type plants

Here, we assume that (for all versions of the model), prior to the initiation of lateral root emergence, no auxin is supplied by the primordia (so that $\omega_{\text{auxin}} = 0$, see (2.21)) and that the system is at steady state (this being the initial condition of the system for all model simulations). This steady state depends in part on the basal auxin production rate $\omega_{\text{auxin}}^{\text{basal}}$ (again see equation (2.21)). In the model, $t = 0$ corresponds to the time at which the LRP begins to supply auxin to the overlying tissues. Thus, for $t > 0$, we set ω_{auxin} to its default (i.e. non-zero) value, this being given in Table M2. The system then tends to its post-initiation steady-state. All (except two) simulations of wild-type plants were performed using the default parameter setting, the exceptions being the simulations illustrated in main text figure 4G and Figures 6B and 6C. In Figure 4G, $m = 1$ (whereas normally $m = 3$), whereas in Figure 6B and 6C (right panels) $\mu_{\text{LAX3}} = 0.001$ (i.e. it is 100 times smaller than its default value and μ_{PIN3}, this reflecting slow induction of LAX3; m and ω_{auxin} are set to their default values).

4.2 Cell division

During stage one of lateral root emergence, the XPP cells that form the primordia undergo several rounds of anticlinal division (i.e. divide so that the division plane is perpendicular to the cells' longest axis, see main text Figure 1B). Thus, in one XPP cell file, up to two cells will divide to form eight, then sixteen, then thirty-two cells. These cells will produce a source of auxin (as measured by DR5 expression [4]). To check to see whether this would influence the behaviour of the model, we ran simulations where several rounds of cell division occurred (see Figure M2) and then compared this to case where no divisions took place. The cell divisions were simulated by updating the tissue geometry each time division a occurred. This also required that, when moving from a pre-division tissue geometry to the post-division one, the ODE solver had to be restarted with new initial conditions. Here, cells that do not divide maintained their pre-division state; for cells that do divide, the daughter cells adopt the states of their parents. The results of the two model simulations (with divisions and with out them) are indistinguishable, this being because the volume of the daughter cells will be contained within the volume of the parent cells, so the nature (from the perspective of the overlying endodermal cells) of the source remains unaltered.

4.3 Auxin dose response experiments

During auxin dose response experiments, roots are treated with exogenous auxin for several hours, and the level of cortical LAX3-YFP fluorescence was measured (see Figure 4H). To model the exogenous source of auxin, we assume that each (externally facing) epidermal apoplast compartment is subject to an additional auxin influx rate. This is assumed to be proportional to the concentration of exogenous auxin and therefore taken to be constant (namely ω_{exo}). We note that (in the model) there are no auxin carriers expressed in the epidermis (this reflecting the expression patterns of known auxin carriers). However, the auxin can diffuse through the epidermis, and accumulate in the cortex. Thus, following Section 2.2.2, the equations governing (externally facing) epidermal apoplast compartments in this special case are:

$$[\text{auxin}]^{\text{apo}}_{l,0} = \frac{B_1 P_{\text{IAAh}}[\text{auxin}]_{l,0} + B_1 P_{\text{IAAh}}\omega_{\text{exo}}}{A_1 P_{\text{IAAh}}}, \tag{4.1}$$

noting that, for $\omega_{\text{exo}} = 0$ (this being its default value), (4.1) satisfies the zero-flux boundary condition (2.24). This approach is adopted for all versions of the model.

We assume that, during the dose response experiments, the system has approached steady state and that the level of LAX3-YFP fluorescence is proportional to the concentration of its protein. In the model, this corresponds to varying ω_{exo} in (4.1) and calculating the steady-state level of LAX3 protein in each cell. However, each cell in the model geometry has (as in reality) a slightly different volume, surface area and so on. Thus, the levels of LAX3 will vary from cell to cell for a given concentration of exogenous auxin. This reflects biological reality; the experimental data presented is also an average over many different cells (and roots), each of which have slightly different levels of LAX3. Thus, we compare the experimental data to $\langle[\text{LAX3}]_j\rangle$ (where angle brackets denote the average over all cortical cells). Plotted in Figure M3 are various simulations of the LAX3 dose response. In the experiments, we find that there is a sigmoidal relationship between exogenous auxin and LAX3-YFP fluorescence levels. To capture this in the model, we find that we must choose m, the Hill coefficient for transcription factor X or ARF7 binding to the LAX3 promoter (see (2.10) and (2.5b)), to be greater than one. The expression of PIN3/AEC causes a shift in the dose response curve, by causing an additional efflux of auxin from the cortical cells (such that higher concentrations of exogeneous auxin are required to counteract its effect), but does not change the overall shape. We have therefore varied the basal level of auxin to compensate for this. The results presented in Figure M3 were generated using the final version of the model (i.e. version 3, where LAX3 is secondary response and PIN3/AEC is primary response). However, as we explain in Section 2.1.1, the steady states are unaltered when LAX3 is considered to be primary response (as is the case in model versions 1 and 2). Thus, the response curves provided in Figure M3 are relevant for all model versions.

4.4 Steady-states for various LRP auxin supply rates (ω_{auxin})

We investigated the sensitivity of the models' behaviour to variations in ω_{auxin} and to whether cells in one XPP or three XPP files provide the auxin source. To do this, we solved the time-dependent models numerically (using MATLAB routine ode15s). The solutions were computed for large enough time intervals that the system reaches equilibrium; from this the steady-state plots presented in Supplementary Modelling Figures M7-M25 were computed. In the case where the system was bistable, steady states were for simplicity computed by starting the system at multiple initial conditions.

4.5 Simulation of NOA and NPA treatments

In this work we also experimentally perturb the system by treating roots with NOA, a chemical that antagonises AUX1/LAX-mediated transport of auxin. To simulate this, we assume that lateral root emergence is initiated prior to NOA treatment, and so at $t = 0$ (when treatment is applied) the system is initially close to its post-initiation steady state (see Section 4.1). For $t > 0$, we set P_{AUX1} and P_{LAX3} to zero. In the data, NOA treatment causes LAX3 expression to spread from just two cells to many, in both circumferential and longitudinal directions. Time-resolved confocal microscopy of the spread clearly shows that it is initiated from cortical cells overlying the primordium and that it moves from one cortical cell to another in a sequential manner (Supplementary Figure S4B and S6C). Given the degree of the spread, it is likely that the NOA treatment is also causing the size of the auxin source (provided by the primordia) to increase; we therefore choose ω_{auxin} (see (2.21)) so that it is 20 times larger than its default value. In the case of model version one (where PIN3 is not included, see Section 2.1.1) LAX3 expression does not spread (see main text Figure 5C and contrast with Figure 5B). If the fold change in auxin supply rate is particularly high (not 20 but 200 fold, for example) then LAX3 expression does spread to one additional file. However, such large fold changes are likely to be unphysiological (i.e. recall that this is 200 fold more auxin than is required to induce LAX3 to 'normal' levels). We next assumed that an auxin inducible auxin efflux carrier was facilitating the spread, and that this carrier was polarized towards neighbouring cortical cells (a natural assumption given the nature and direction of the spread). Upon simulating the NOA experiment for this case, we observed LAX3 would spread to multiple cells (unlike in model version one). However, the model could not capture the extent of the spread observed experimentally. We therefore again assumed that the size of the source was also being effected by the NOA treatment; in this case (by again assuming the auxin source increased by a factor of 20) LAX3 was predicted to spread to all cortical cells (as observed experimentally, main text Figure 5D).

5 Analytical treatment of the steady-state problem

Here we discuss analytical solutions to the steady-state problem for model version one. Our numerical analysis of this model version (see Supplementary Modelling Figures M7-M13) indicated that it is unable to account for the available experimental data, namely that LAX3 expression is restricted to one or two cell files (see main text and Figure 1 for details). In our simulations, LAX3 is expressed in almost every cortical cell that makes (indirect) contact with an XPP source cell, of which there are typically several. However, of these cortical cell files, there are two major ones (for each XPP source cell file) which share the majority of the surface area with the adjoining endodermal cells. The other ones are referred to as "minor" cortical files. If the model is to account for our observation that LAX3 is only expressed in at most two cell files (main text Figure 1), then we expect this expression will occur only in the major but not the minor files. This suggests that the LAX3 steady-state response function (i.e. how LAX3 expression levels vary according to the concentration of intracellular auxin), must be sufficiently sharp. To understand this better, we exploit the limit where carrier-mediated transport of auxin dominates over passive diffusion in governing its movement between cells and the apoplast (whereby $A_1 \ll 1$, $A_3 \ll 1$, $B_1 \ll 1$, $B_2 \ll 1$, which are all small in practice, see Table M2). We then use this to obtain conditions on how sharp the LAX3 steady-state response curve should be for the model to be consistent with the data (see in particular main

text Figure 1E).

More formally, we use asymptotic methods to derive approximate expressions for the steady-state solutions of the full model. To do this we let $\epsilon = A_1$, and rescale parameters A_3, B_1, B_2 by ϵ so that $A_3 = \epsilon\bar{A}_3$ etc and then take the limit $\epsilon \to 0$.

5.1 Auxin supplied by the LRP

For the case where the auxin source is supplied by the LRP, we assume that in the limit $\epsilon = A_1 \to 0$, the auxin source provided by the XPP cells is large enough for it to still enter the endodermis (so that $\omega_{\rm auxin} A_1 = \omega_{\rm auxin}\epsilon = O(1)$ as $\epsilon \to 0$). Thus, we rescale $\omega_{\rm auxin}$ by $1/\epsilon$. We will find that by taking this limit, we can derive approximations to the full model which are very accurate (see Figure M4) whilst now being amenable to mathematical analysis. It follows from (2.18) that, in the limit $\epsilon \to 0$, the auxin fluxes between neighbouring cortical cells i, j and their adjoining apoplast compartment are approximately

$$J_{{\rm IAAh}_{{\rm Cor}i,{\rm Cor}j}} + J_{{\rm LAX3}_{{\rm Cor}i,{\rm Cor}j}} \sim P_{\rm LAX3}[{\rm LAX3}]_{{\rm Cor}i} A_2 [{\rm auxin}]_{{\rm Cor}i,{\rm Cor}j}, \tag{5.1}$$

$$J_{{\rm IAAh}_{{\rm Cor}j,{\rm Cor}i}} + J_{{\rm LAX3}_{{\rm Cor}j,{\rm Cor}i}} \sim P_{\rm LAX3}[{\rm LAX3}]_{{\rm Cor}j} A_2 [{\rm auxin}]_{{\rm Cor}j,{\rm Cor}i}, \tag{5.2}$$

$$\tag{5.3}$$

where we write Cor to emphasize cell type. However, it follows from (2.23) that the steady-state concentration of auxin in the adjoining apoplast compartment ($[{\rm auxin}]_{{\rm Cor}i,{\rm Cor}j}$) is zero at $O(1)$ (noting that $[{\rm PIN3}] \equiv 0$ since there is no PIN3 in this model). Thus, auxin fluxes between neighbouring cortical cells are negligible, corresponding to the case where there is little cell-cell communication. Similar results hold for fluxes between cortical and epidermal cells. The flux into cortical cell i from the apoplast compartment connecting it to endodermal cell k is given by

$$J_{{\rm IAAh}_{{\rm Cor}i,{\rm En}k}} + J_{{\rm LAX3}_{{\rm Cor}i,{\rm En}k}} \sim P_{\rm LAX3}[{\rm LAX3}]_{{\rm Cor}i} A_2 [{\rm auxin}]_{{\rm En}k,{\rm Cor}i}, \tag{5.4}$$

and the steady state concentration of auxin in the adjoining apoplast compartment ($[{\rm auxin}]_{{\rm En}i,{\rm Cor}j}$) is

$$[{\rm auxin}]_{{\rm En}k,{\rm Cor}i} = \frac{P_{\rm UEC} B_3 \omega}{P_{\rm LAX3}[{\rm LAX3}]_{{\rm Cor}i} A_2}, \tag{5.5}$$

where ω is the concentration of auxin in the adjoining endodermis. In the full model, this will depend on the strengths of the various carriers (in particular UEC) transporting the auxin from the source XPP cell. Collecting (5.4)-(5.5) together and substituting them into (2.22), we obtain the steady-state concentration of auxin in a cortical cell (for a given concentration of auxin in the connecting endodermal cell), namely:

$$[{\rm auxin}]_{{\rm Cor}i} = \sum_k \Psi_{i,k}\omega + \frac{\omega^{\rm basal}_{\rm auxin}}{\mu_{\rm auxin}}, \tag{5.6}$$

where we are summing over all endodermal cells k which directly contact cortical cell i, and

$$\Psi_{i,k} = \frac{B_3 P_{\rm UEC} S^{{\rm Cor}i,{\rm En}k}}{V_{{\rm Cor}i}\mu_{\rm auxin}}, \tag{5.7}$$

where $V_{{\rm Cor}i}$ is the volume of cortical cell i and $S^{{\rm Cor}i,{\rm En}k}$ is the surface area connecting it to endodermal cell k. Note that terms pertaining directly to LAX3 do not appear in (5.6). We can write steady-state

level of LAX3 in cell i explicitly as a function of intracellular auxin, namely:

$$[\text{LAX3}]_{\text{Cor}i} = L([\text{auxin}]_{\text{Cor}i}), \tag{5.8a}$$

and L can be obtained directly by solving the steady-state problem to (2.5)-(2.7) (for which there is only one positive solution: i.e. the model is monostable and not bistable, as is the case for some parameter sets in model version two/three). Thus, the (approximate) steady-state concentration of LAX3 in cortical cell i is given by

$$[\text{LAX3}]_{\text{Cor}i} = L\left(\sum_k \Psi_{i,k}\omega + \frac{\omega_{\text{auxin}}^{\text{basal}}}{\mu_{\text{auxin}}}\right). \tag{5.9}$$

Comparisons between the full model steady-state solutions together with the approximated solutions (5.9) are provided in Figure M4.

5.2 Exogenous auxin treatment

To obtain approximate solutions to the steady-state when the level of exogenous auxin is being varied (as is the case in the auxin dose response experiments), we assume that the source of auxin is large enough so that auxin can enter the cortex from the epidermis. Let ω_{exo} be the epidermal concentration of auxin so that $\omega_{\text{exo}}B_1 = O(1)$. We can then apply similar arguments to obtain:

$$[\text{LAX3}]_{\text{Cor}i} = L\left(\sum_k \frac{\omega_{\text{exo}}}{\Psi_{i,k}^{\text{exo}}} + \frac{\omega_{\text{auxin}}^{\text{basal}}}{\mu_{\text{auxin}}}\right), \tag{5.10}$$

where

$$\Psi_{i,k}^{\text{exo}} = \frac{B_1 P_{\text{IAAh}} S^{\text{Cor}i,\text{Ep}k}}{V_{\text{Cor}i}\mu_{\text{auxin}}}, \tag{5.11}$$

where Epk denotes epidermal cell k. Plotted in Figure M5 are comparisons between steady-state response of LAX3 (averaged over many cells), obtained from either the full model or the approxmation given by (5.10).

5.3 Conditions on the sharpness of the LAX3 steady-state response function

The function L in (5.8) depends on a number of parameters reflecting interactions with the LAX3 regulatory network (see Section 2.1.1), and these will determine its shape. However, we know from Section 5.2 that this shape can be obtained directly by measuring the LAX3 response to exogenous auxin treatment (as shown in main text Figure 4H). We can approximate its shape using a Hill function: this is convenient analytically, and it allows us to compare our results directly to the available dose response data (main text Figure 4H; we note that usage of the more complex (and detailed) gene network model, as described in Section 2.1.1, is useful for understanding the time-dependent problem in LAX3 induction). We therefore write:

$$L([\text{auxin}]) = \frac{[\text{auxin}]^h}{\theta^h + [\text{auxin}]^h}, \tag{5.12}$$

where θ give the concentration of auxin at which LAX3 is half-maximal, and h is the effective Hill coefficient (note that h and m in (2.5) are not necessarily equal). We then consider two cells, where cell one is in a major cortical cell file, and cell two is in a minor cell file, both connecting to the same endodermal file, so that $S^{\mathrm{Cor1}} > S^{\mathrm{Cor2}}$. We are interested in steady-state solutions to model version one auxin levels are such that the expression of LAX3 expression in the major cortical cell is at 50% maximum, and LAX3 is expressed at a low level in the minor cell file. Furthermore, we require that this pattern of expression is robust to variations of ω by up to $\alpha \times 100\%$. Upon substituting the constraints into (5.9) for cell one, we obtain the following mathematical expression of the level of LAX3 in cell two:

$$[\mathrm{LAX3}]_{\mathrm{Cor2}} = \frac{\left(\alpha(1+\rho) + \left(\frac{[\mathrm{LAX3}]_{\mathrm{basal}}}{1-[\mathrm{LAX3}]_{\mathrm{basal}}}\right)^{\frac{1}{h}} (1-\alpha(1+\rho))\right)^h}{1 + \left(\alpha(1+\rho) + \left(\frac{[\mathrm{LAX3}]_{\mathrm{basal}}}{1-[\mathrm{LAX3}]_{\mathrm{basal}}}\right)^{\frac{1}{h}} (1-\alpha(1+\rho))\right)^h}. \tag{5.13}$$

where $[\mathrm{LAX3}]_{\mathrm{basal}}$ is the level of LAX3 when auxin are at basal levels and $\alpha = S_2/S_1$, the ratio between the shared surface areas of the minor and major cell files (see Supplementary Figure 3). Plotted in Figure M6 are solutions to (5.13) for fixed $[\mathrm{LAX3}]_{\mathrm{Cor2}}$ (i.e. the level of expression in the minor cell file) and various h and α. Here, we set $[\mathrm{LAX3}]_{\mathrm{basal}} = 0.01$, using the level of LAX3 measured in untreated roots (Figure 4H) for an rough order-of-magnitude estimate (since this measurement is also likely to contain some background noise). As noted in the main text α is on average 1/3, and so even if we allow that $[\mathrm{LAX3}]_{\mathrm{Cor2}}$ is 0.1 (one-fifth the level in the major cortical cell file, which in this case is fixed to 0.5), then the effective Hill coefficient h would have to be at least 4 (twice that was measured, see Figure 4H). If LAX3 in the neighbouring major cortical cell file were larger than 0.5, or if $\alpha > 1/3$, then the effective Hill coefficient would have to be even larger to compensate.

6 Hill function fits

The auxin-dose response data for LAX3-YFP, presented in main text Figure 4H, was fitted to a Hill function, namely

$$g(y) = \frac{y^p}{y^p + \theta^p}, \tag{6.1}$$

where p and θ are respectively the Hill coefficient and the threshold. To do this, we used the cost function

$$f = \sum_{i=1}^{n} \frac{(\mathrm{LAX3}_i - g(A_i))^2}{\sigma_i^2}, \tag{6.2}$$

where the sum is over all n data points. Here, σ_i, A_i and $\mathrm{LAX3}_i$ are respectively the standard deviation, the concentration of exogenous auxin, and the level of LAX3-YFP florescence detected (normalised so that it has a maximum level of one) for the ith data point. Estimates for p and θ were obtained by minimizing the cost function f (6.2) using MATLAB routine `fminsearch`. The best fit is provided in main text figure 4H, for which the effective Hill coefficient $p = 2$ and the threshold $\theta = 40$nM.

Reference	Parameter	Default value	Meaning & comments
Equation (2.17)	A_1	0.0594	Proportion of protonated auxin in the cell wall
Equation (2.19)	A_2	4.4088	Proportion of anionic auxin in the cell wall which is transported by LAX3
Equation (2.19)	A_3	0.0425	Proportion of anionic auxin in the cell wall which is transported by PIN3
Equation (2.17)	B_1	0.004	Proportion of protonated auxin in the cytoplasm
Equation (2.19)	B_2	0.045	Proportion of anionic auxin in the cytoplasm which is transported by LAX3
Equation (2.19)	B_3	4.68	Proportion of anionic auxin in the cytoplasm which is transported by PIN3
[27]	Pk	4.8	Acid dissociation constant for auxin
[27]	$\mathrm{P}^{\mathrm{cyt}}$	7.2	Cytoplasm pH
[7]	$\mathrm{P}^{\mathrm{apo}}$	6	Apoplast compartment pH
[27]	ϕ	-4.64	Dimensionless constant
[27]	P_{IAAh}	0.5 $\mu\mathrm{ms}^{-1}$	Passive membrane permeability
[27]	P_{AUX1}	0.5 $\mu\mathrm{ms}^{-1}$	Membrane permeability due to AUX1
[27]	P_{LAX3}	0.5 $\mu\mathrm{ms}^{-1}$	Membrane permeability due to LAX3
[27]	P_{PIN3}	0.5 $\mu\mathrm{ms}^{-1}$	Membrane permeability due to PIN3
[27]	P_{UEC}	0.5 $\mu\mathrm{ms}^{-1}$	Membrane permeability due to the unidentified efflux carrier
[16]	λ	0.1 μm	Apoplast thickness
-	ω_{auxin}	0.3 min^{-1}	production rate of auxin by the primordia
-	$\omega_{\mathrm{auxin}}^{\mathrm{basal}}$	10^{-5} min^{-1}	basal production rate of auxin by the primordia
-	μ_{auxin}	1 min^{-1}	decay rate of auxin
-	μ_{PIN3}	0.1 min^{-1}	decay rate of auxin efflux carrier PIN3
-	μ_{LAX3}	0.1 min^{-1}	decay rate of auxin efflux carrier LAX3
-	μ_{X}	0.1 min^{-1}	decay rate of transcription factor X
-	l_m	1 min^{-1}	maximal decay rate of IAA14
-	δ_{IAA14}	1 min^{-1}	translation rate of *IAA14* mRNA
-	θ_{PIN3}	0.1	binding threshold for ARF7 to PIN3 promoter
-	θ_{IAA14}	0.1	binding threshold for ARF7 to IAA14 promoter
-	θ_{X}	0.1	binding threshold for ARF7 to transcription factor X promoter
-	ψ_{PIN3}	0.1	binding threshold for IAA14-ARF7 to PIN3 promoter
-	ψ_{IAA14}	1	binding threshold for IAA14-ARF7 to IAA14 promoter
-	ψ_{X}	0.1	binding threshold for IAA14-ARF7 to transcription factor X promoter
-	θ_{LAX3}	0.1	binding threshold for X to LAX3 promoter
-	ϕ_{LAX3}	1	rate of *LAX3* translation to rate of protein LAX3 decay ratio
-	ϕ_{PIN3}	1	rate of *PIN3* translation to rate of protein PIN3 decay ratio
-	ϕ_{X}	1	rate of X translation to rate of protein X decay ratio
-	[ARF7]	1	level of ARF7
-	m	3	Hill coefficient for either factor X or ARF7 binding to the LAX3 promoter (model version 3 and 1,2 respectively)

Table M2: Default values for the parameters in each of the model versions. Quantities are dimensionless unless otherwise stated.

7 Supplementary Modelling Figures

7.1 Additional results and information (Figures M1-M6)

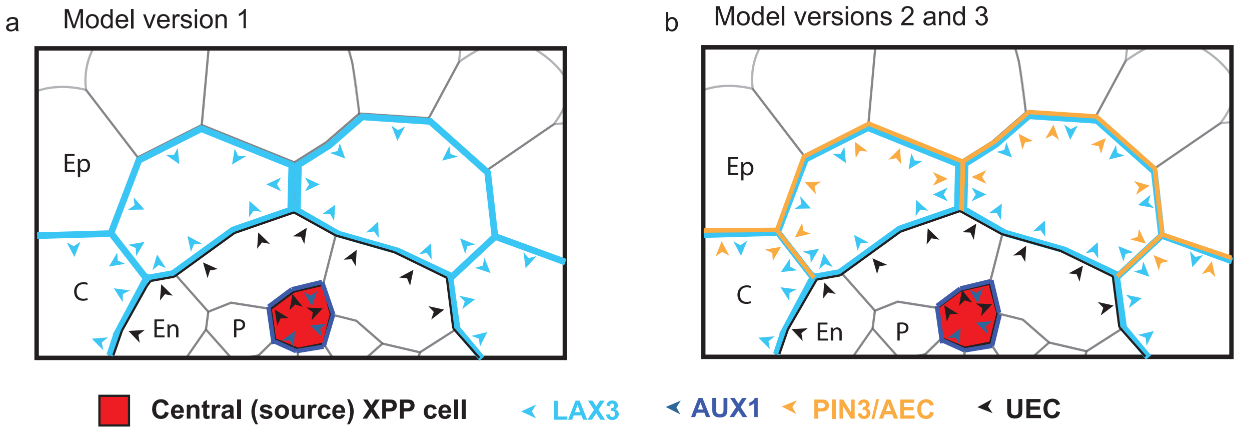

Figure M1: Schematic representation of auxin transport in the three model versions, showing the spatial localisation of the various carriers (this being reflected by our choice of $\Phi^{\chi}_{l,k}$ parameters in the model, see Section 2.2.2). We note that in the model *LAX3* and *PIN3/AEC* are auxin inducible, whereas UEC and AUX1 are assumed to be constitutively expressed. Furthermore, initially for model version two we assumed that PIN3/AEC is polarised only towards neighbouring cortical cells - whereas we subsequently find that PIN3 is also polarised towards the epidermis. Arrows indicate the direction in which the various carrier species act. (a) Model version one (see Section 2.1.1) does not contain PIN3/AEC. (b) Model versions two (Section 2.1.2) and three (Section 2.1.3) contain polar PIN3 in the cortex (as is observed experimentally). **P**: pericycle, **En**: endodermis, **C**: cortex, **Ep**: epidermis.

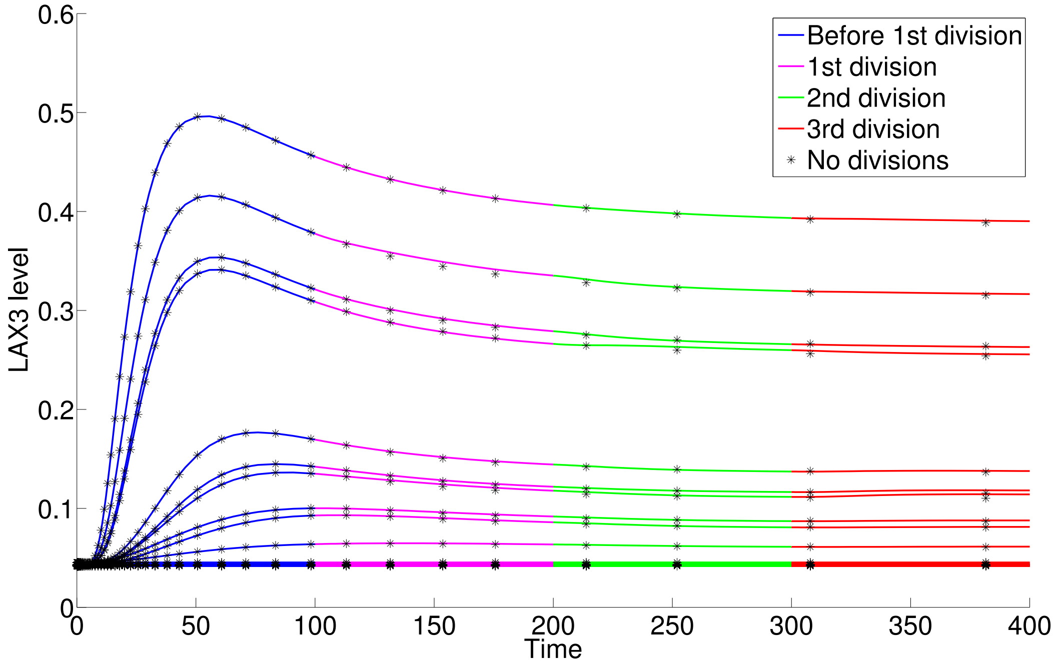

Figure M2: Simulation of model version one with and without cell divisions in the lateral root primordia (a subset of XPP cells). Depicted are changes LAX3 expression levels in various cells over time. See Section 4.2 for more details.

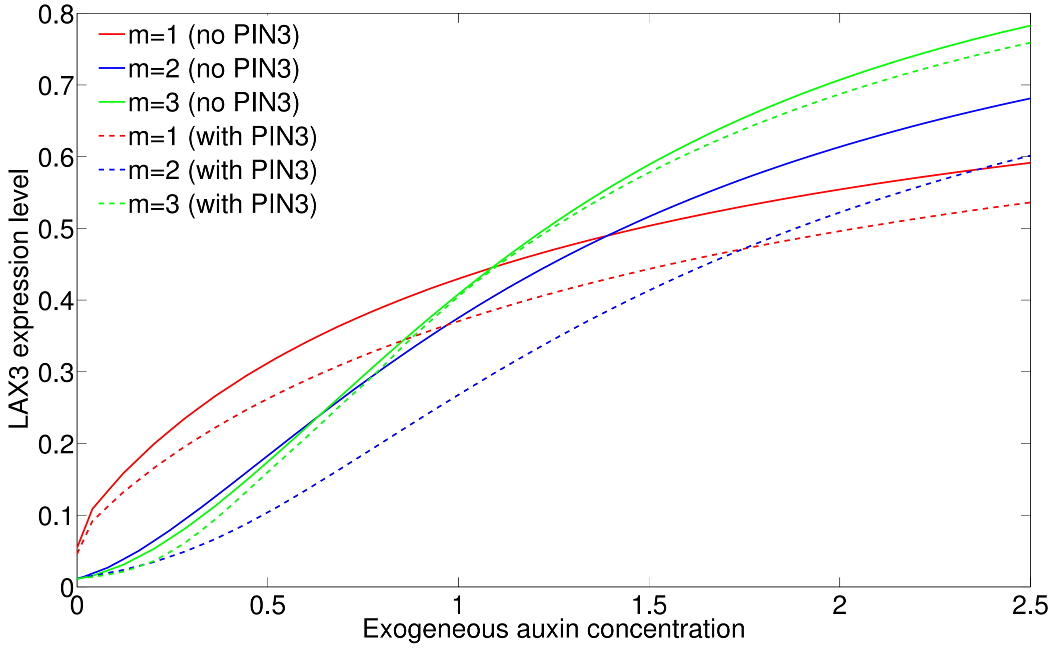

Figure M3: Simulated auxin dose response for LAX3 with and without expression of *PIN3/AEC* and for various m (see Section 4.3 for more details). Plotted is the steady-state level of LAX3 protein (averaged across all cortical cells in the model and over the three different root geometries used) for a given ω_{exo} (see equation (4.1)). For comparison purposes, the basal level of auxin has been chosen so that all model versions express LAX3 at approximately the same level prior to treatment. In case where *LAX3* is modelled as a secondary response gene (as is the case in model version 3), m represents the Hill coefficient for transcription factor X interactions on the LAX3 promoter (see Section 2.1.3 and in particular equation (2.10)). When *LAX3* is modelled as a primary response gene (as is the case in model version 1 and 2), m represents the number of ARF binding sites (see Section 2.1.2). Here, m does not necessarily equal p (the effective Hill coefficient measured in main text Figure 4H, see also Section 6). For $m = 3$, the LAX3 response resembles a Hill function with Hill coefficient ~ 2, whereas for $m = 2$ it has an effective Hill coefficient of ~ 1.5 (this being measured by calculating the gradient of $\log([\text{LAX3}]/(1 - [\text{LAX3}]))$ when plotted against $\log(\omega_{\text{exo}})$). Furthermore, we note that parameters have been chosen so that the steady states for model versions 2 and 3 are the same (again, see Section 2.1.2), and so the dose-response curves for which PIN3/AEC is present are valid for both versions of the model. The response made in model version 1 (Section 2.1.1) corresponds to the solutions where no PIN3/AEC is present.

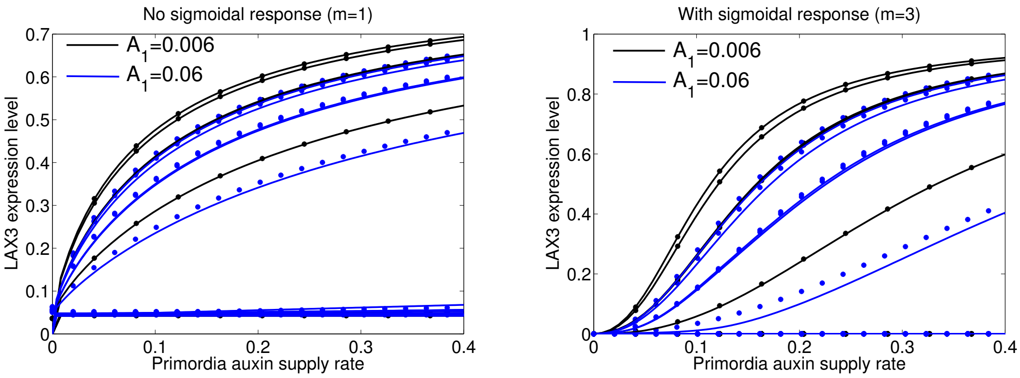

Figure M4: Comparison between full model steady-state solutions (solid lines) and approximate ones (dots; calculated using (5.9)) for two different values of $\epsilon = A_1$ (note that $A_1 = 0.06$ corresponds to its default value, see Table M2) when auxin is being supplied by the LRP. The approximation leads to a very small error. See Section 5 for further details.

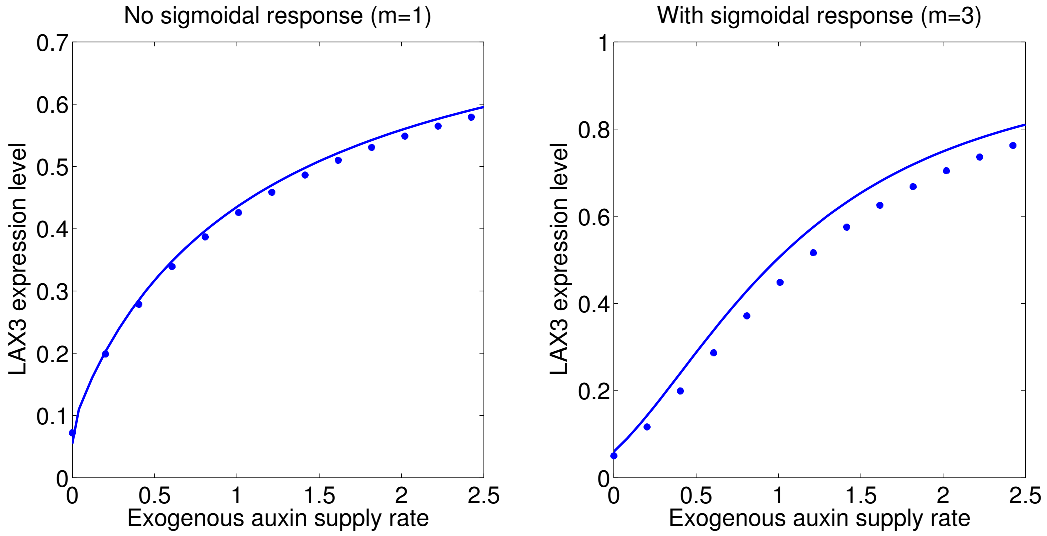

Figure M5: Comparison between full model steady-state solutions (solid lines) and approximate ones (dots; calculated using (5.10)) averaged over all cells for root one. See Figure M3 for more examples of the LAX3 steady-state response to exogenous auxin. Default parameters were used (see Table M2). See Section 5 for further details.

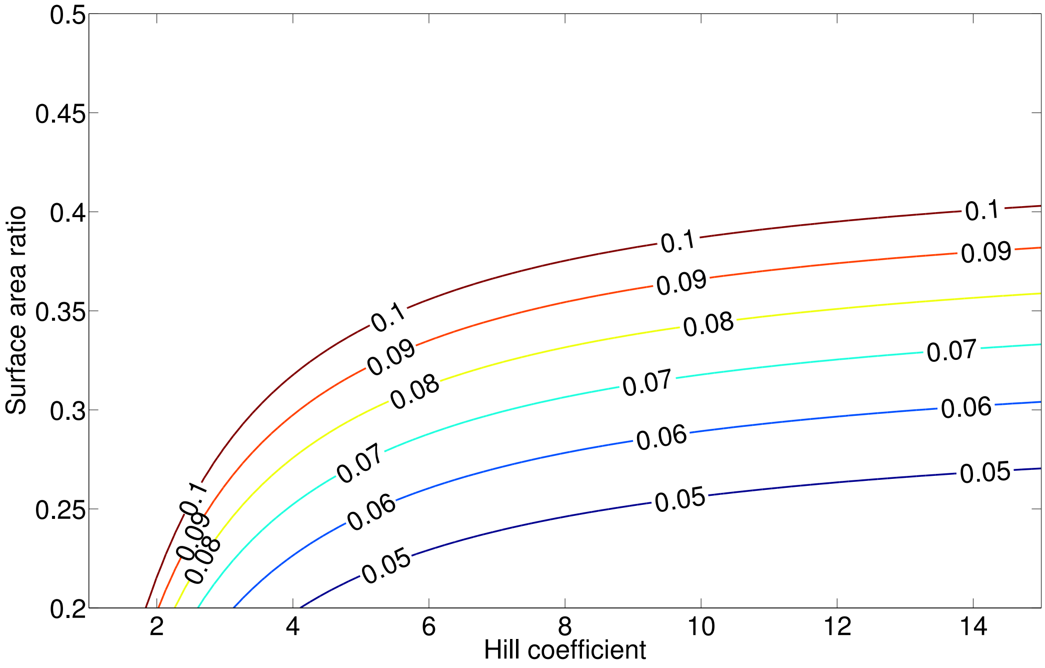

Figure M6: Solutions to (5.13) for various values of $[\mathrm{LAX3}]_{\mathrm{Cor2}}$ plotted against h and α. See Section 5 for further details.

7.2 Steady-state plots (Figures M7-M25)

In this section we plot the steady-state concentration of LAX3 against the rate of auxin supply by the primordium. This is done for each model variants (see Table I for a full overview), each geometry used, and where indicated we have assumed that either the auxin is being supplied by just one XPP cell file or by three. In the plots, colours denote which file a cell is in; dashed (dot-dashed etc) lines are used to distinguish two or more cells that are in the same file (and are therefore plotted using the same colour). We also supply visualisations of the steady-state LAX3 expression pattern for a given level of auxin supply rate (with coloured dots being used to indicate the level chosen). When the LAX3 pattern spans more than one cell per file, we also depict the full three-dimensional pattern, but where the epidermis has been removed.

7.3 Simulations with no sigmoidal response

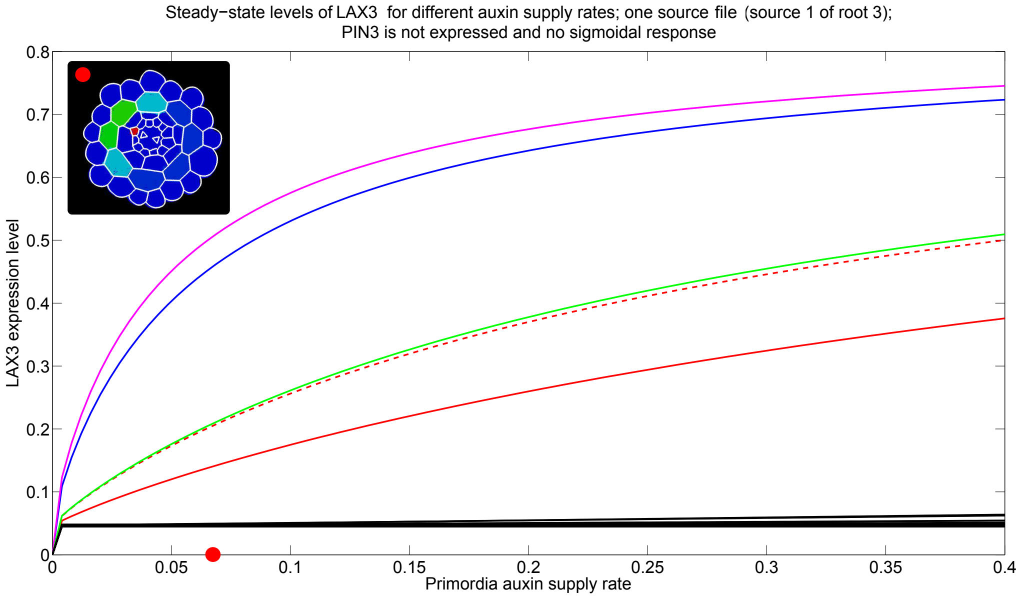

Figure M7: Simulations for root 3 with a single XPP file supplying the auxin source and no PIN3 and no sigmoidal response. Coloured dots indicate level of auxin supply used for the model solution depicted.

For all subsequent simulations, it is assumed that LAX3 gives a sigmoidal response to auxin.

7.4 Simulations for model version one (no PIN expression)

7.4.1 One auxin source file

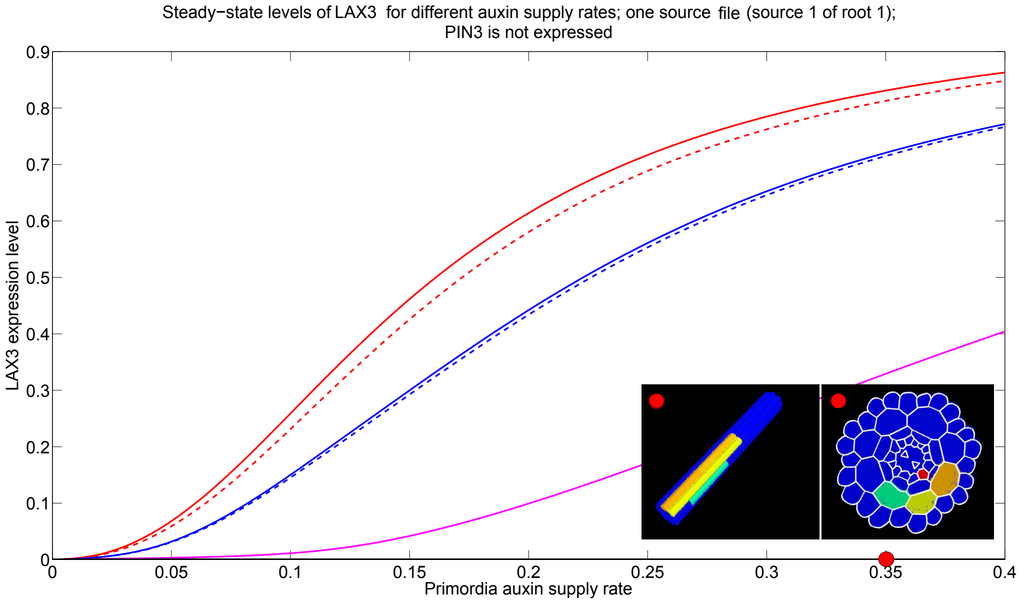

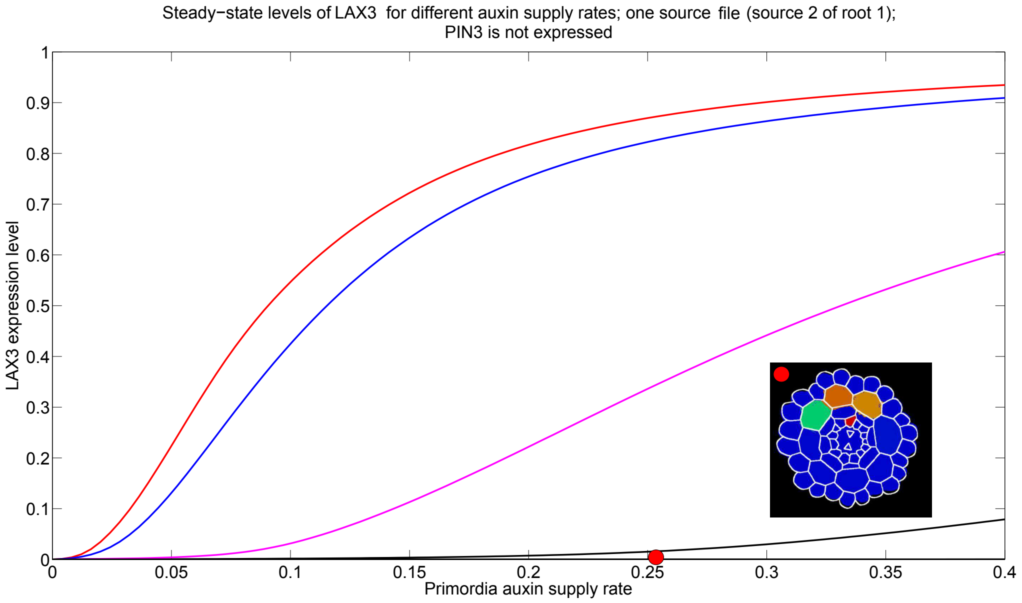

Figure M8: Simulations for root 1 with a single XPP file supplying the auxin source and no PIN3. Colours denote cell file; dashed lines are used to distinguish different cells from the same file. Coloured dots indicate level of auxin supply used for the model solution depicted.

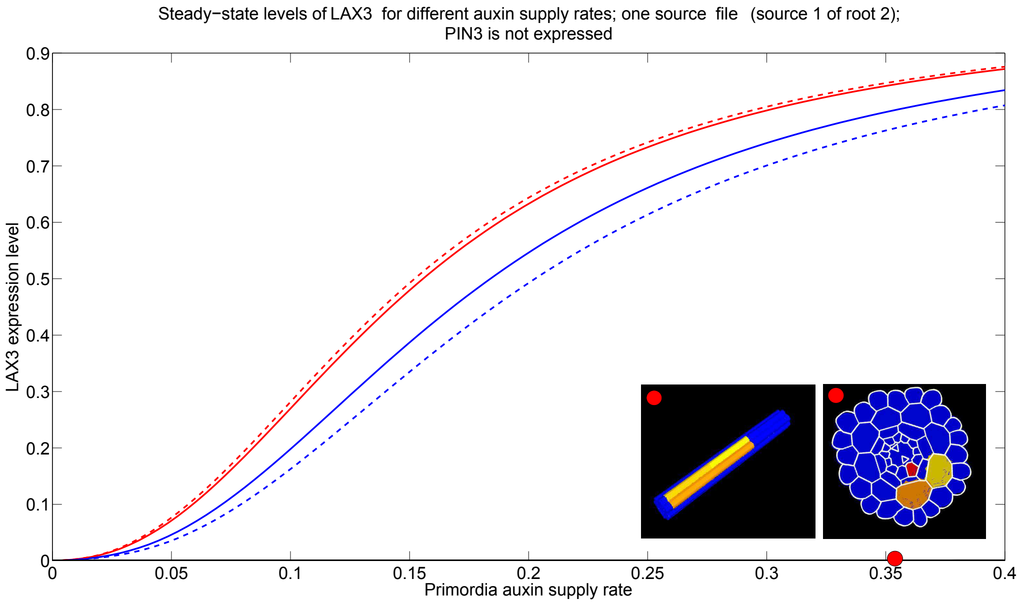

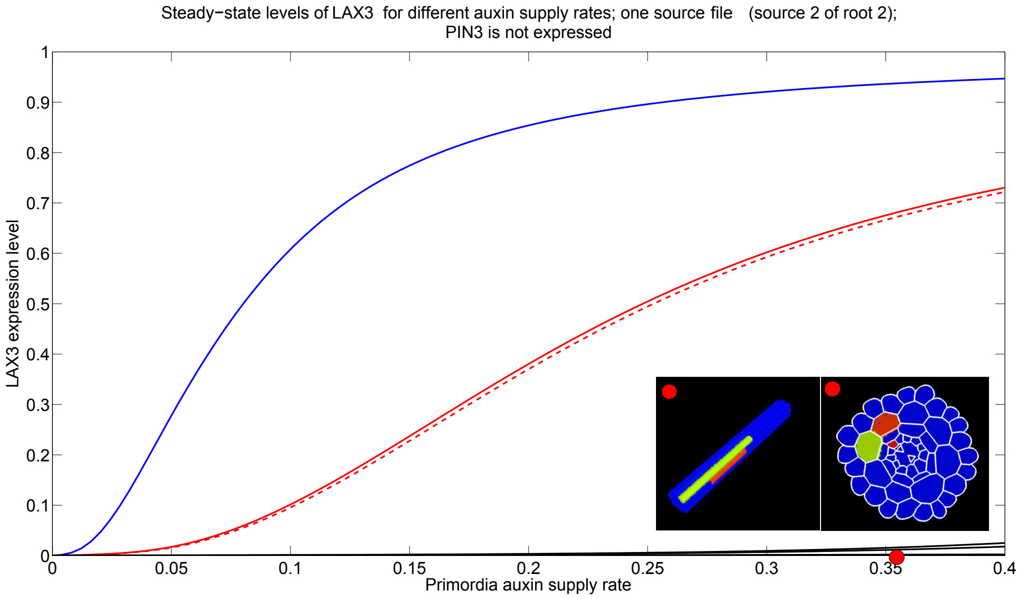

Figure M9: Simulations for root 2 with a single XPP file supplying the auxin source and no PIN3. Colours denote cell file; dashed lines are used to distinguish different cells from the same file. Coloured dots indicate level of auxin supply used for the model solution depicted.

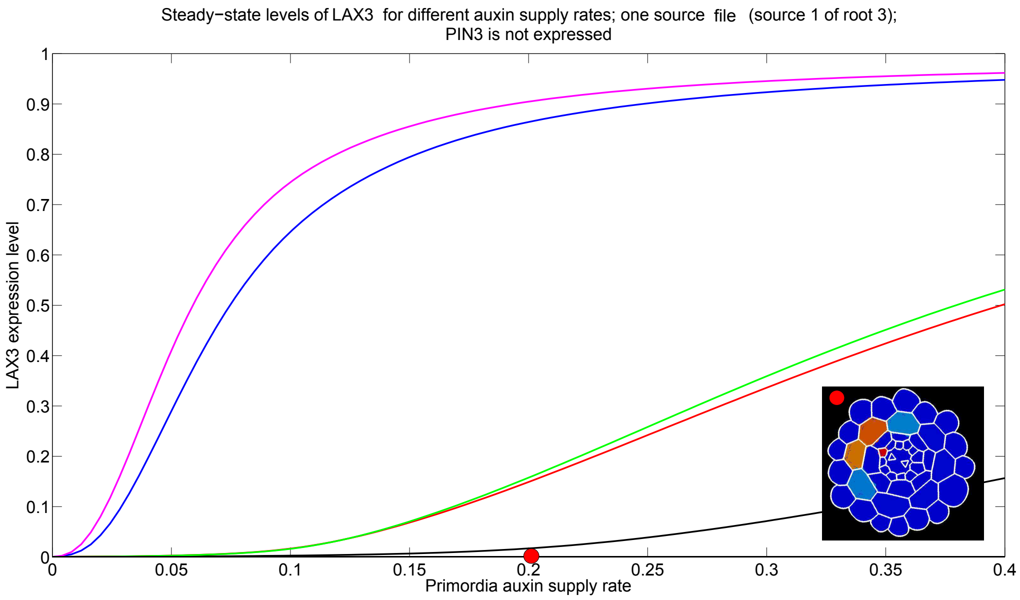

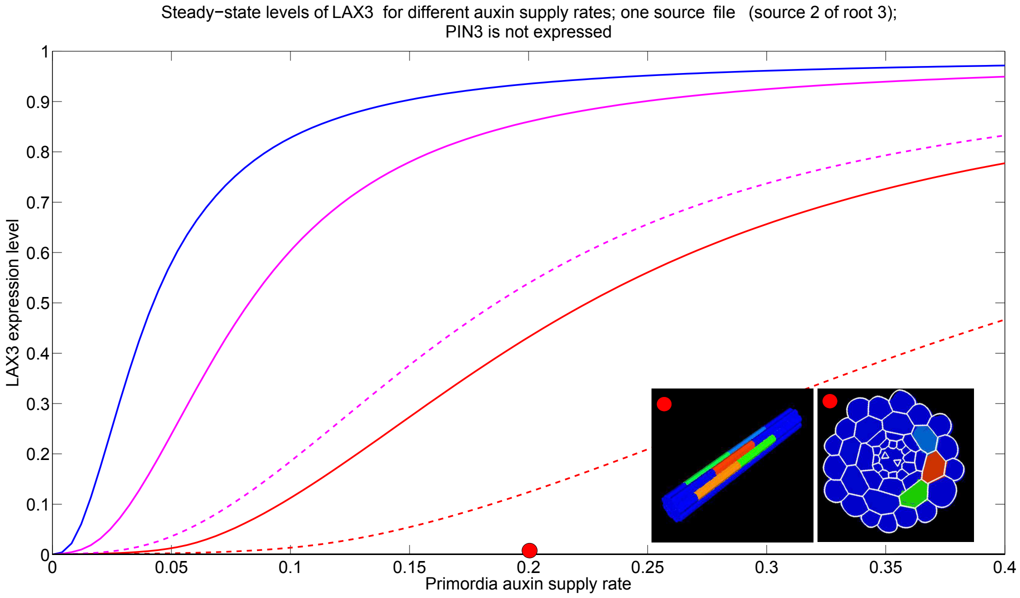

Figure M10: Simulations for root 3 with a single XPP file supplying the auxin source and no PIN3. Colours denote cell file; dashed lines are used to distinguish different cells from the same file. Coloured dots indicate level of auxin supply used for the model solution depicted.

7.4.2 Three auxin source files

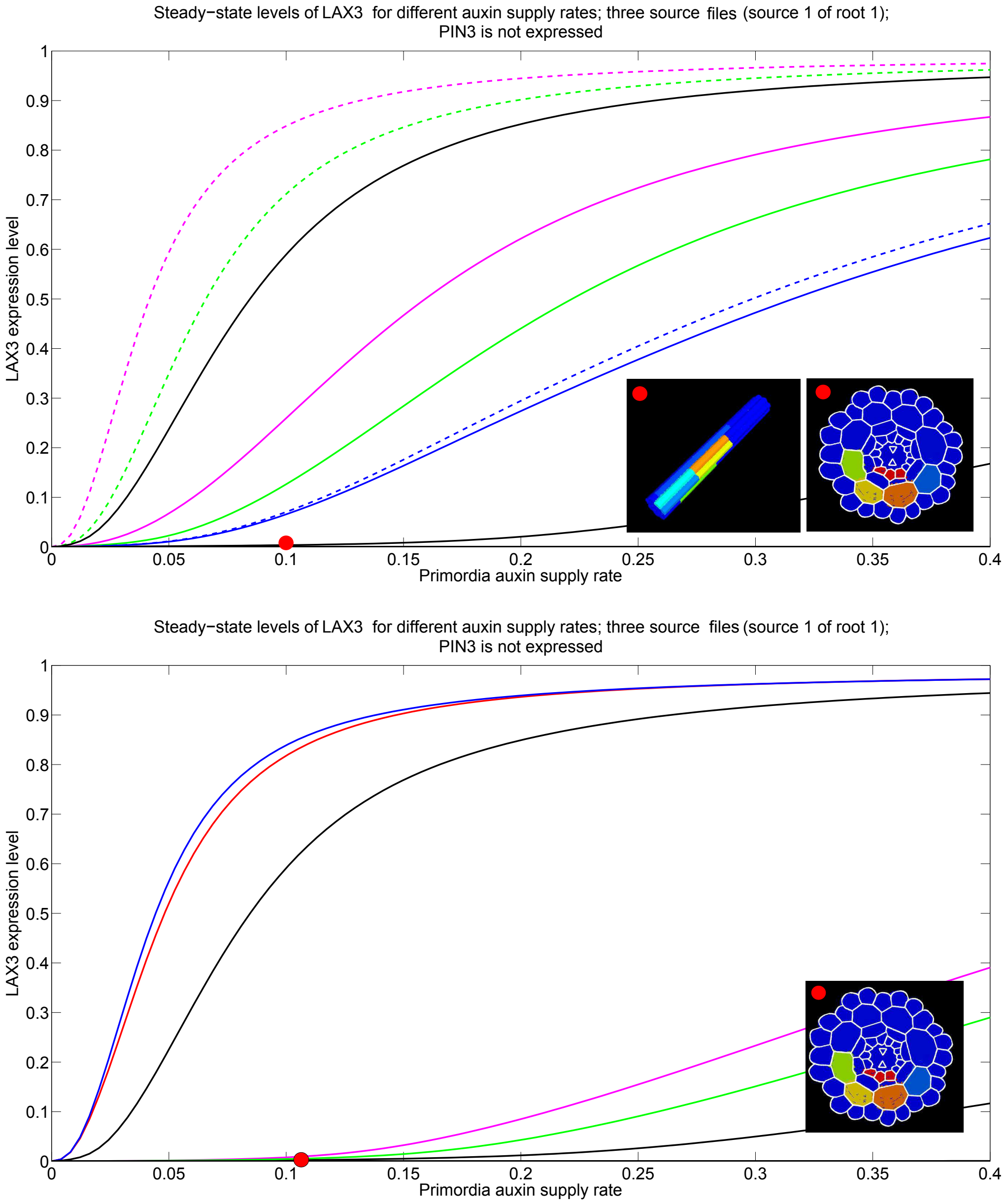

Figure M11: Simulations for root 1 with three XPP cell files acting as a source of auxin and no PIN3. Colours denote cell file; dashed lines are used to distinguish different cells from the same file. Coloured dots indicate level of auxin supply used for the model solution depicted.

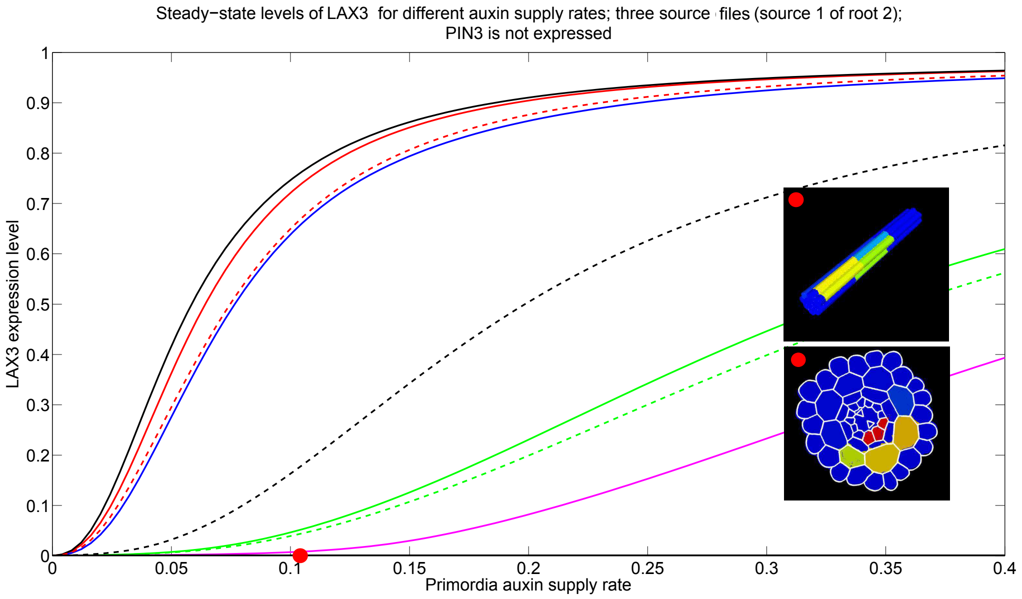

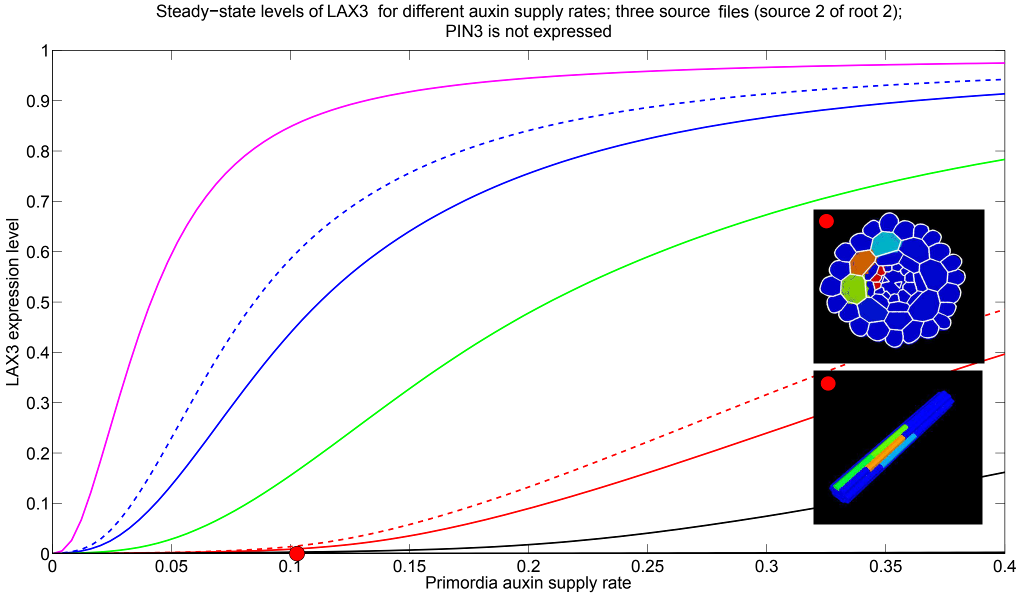

Figure M12: Simulations for root 2 with three XPP cell files acting as a source of auxin and no PIN3. Colours denote cell file; dashed lines are used to distinguish different cells from the same file. Coloured dots indicate level of auxin supply used for the model solution depicted.

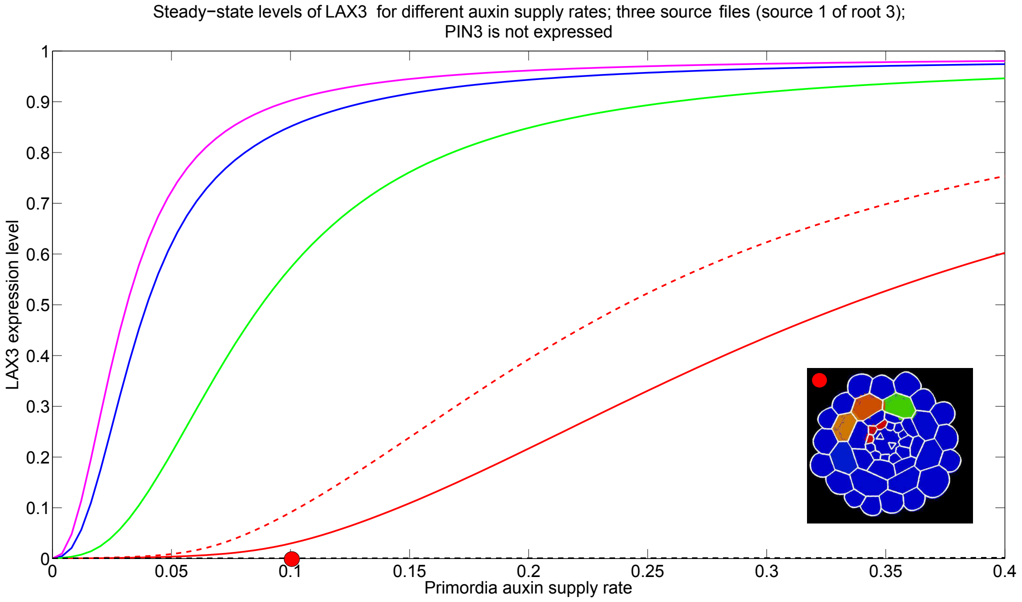

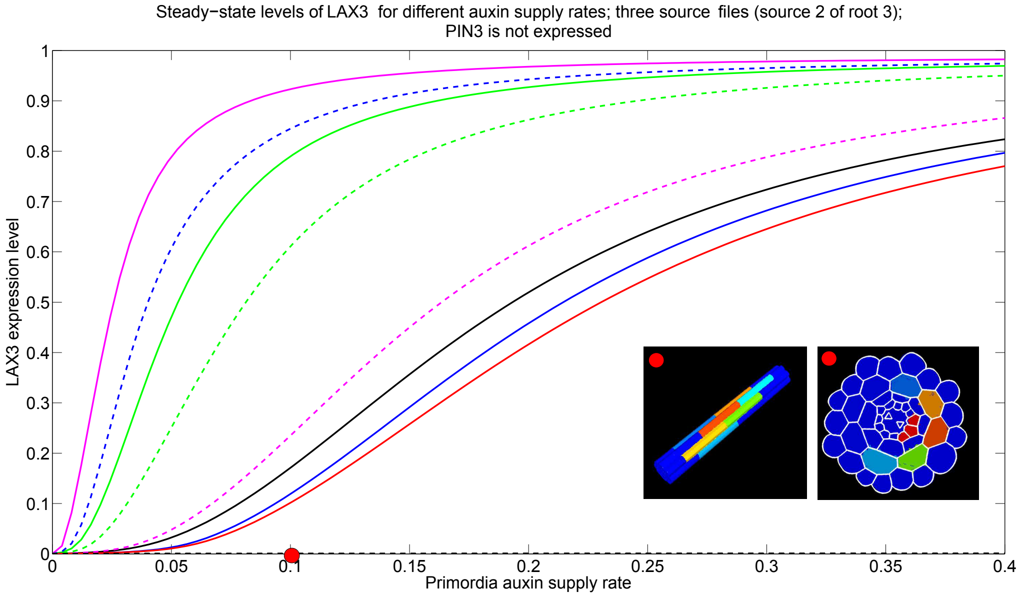

Figure M13: Simulations for root 3 with three XPP cell files acting as a source of auxin and no PIN3. Colours denote cell file; dashed lines are used to distinguish different cells from the same file. Coloured dots indicate level of auxin supply used for the model solution depicted.

7.5 Simulatons for model version two and three (with PIN1 expression)

7.5.1 One source file

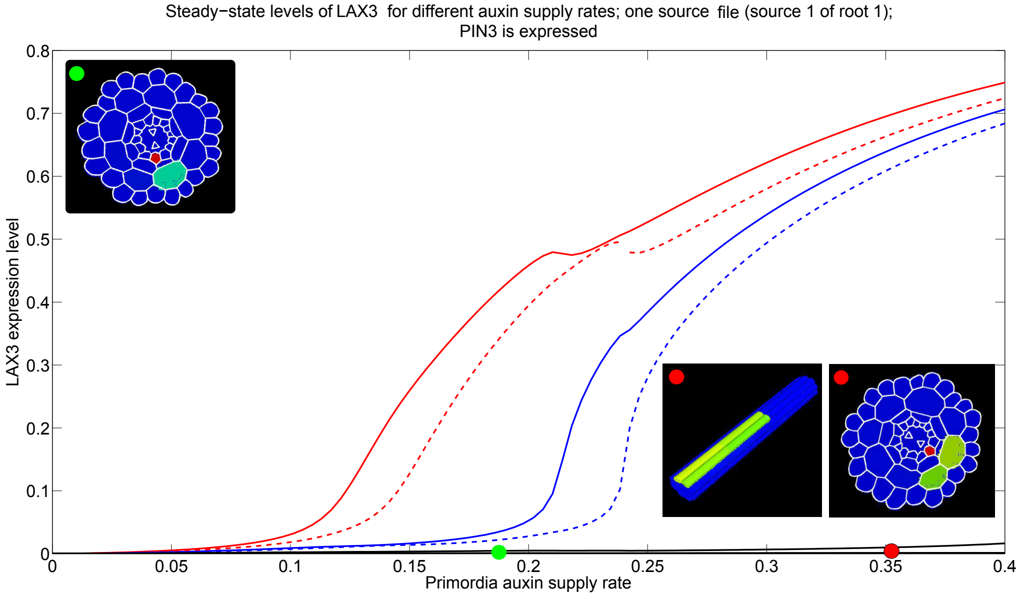

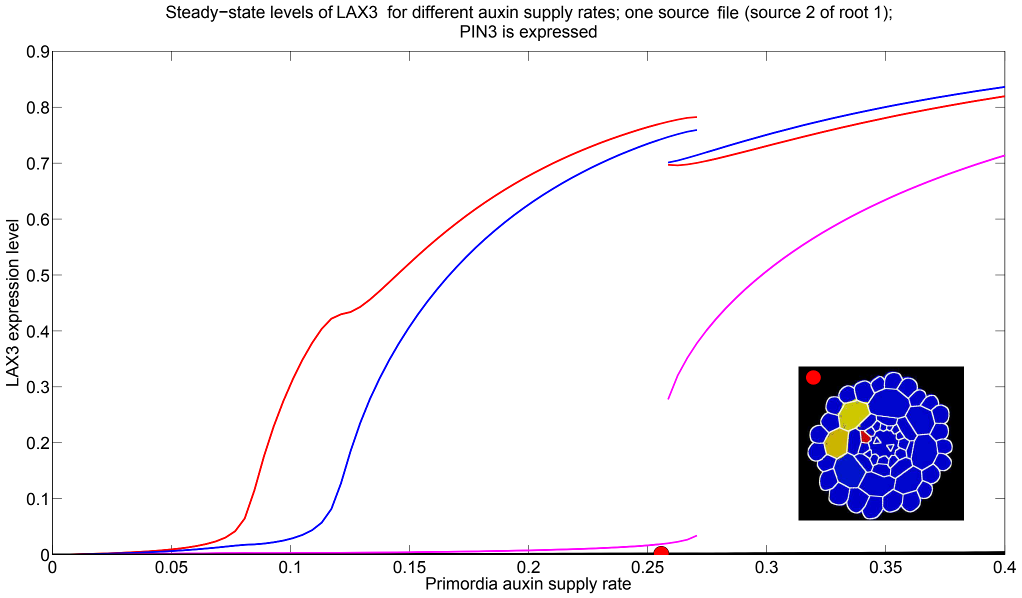

Figure M14: Simulations for root 1 with one XPP cell file acting as a source of auxin and PIN3 is expressed. Colours denote cell file; dashed lines are used to distinguish different cells from the same file. Discontinuities indicate the existence of a bifurcation. Coloured dots indicate level of auxin supply used for the model solution depicted.

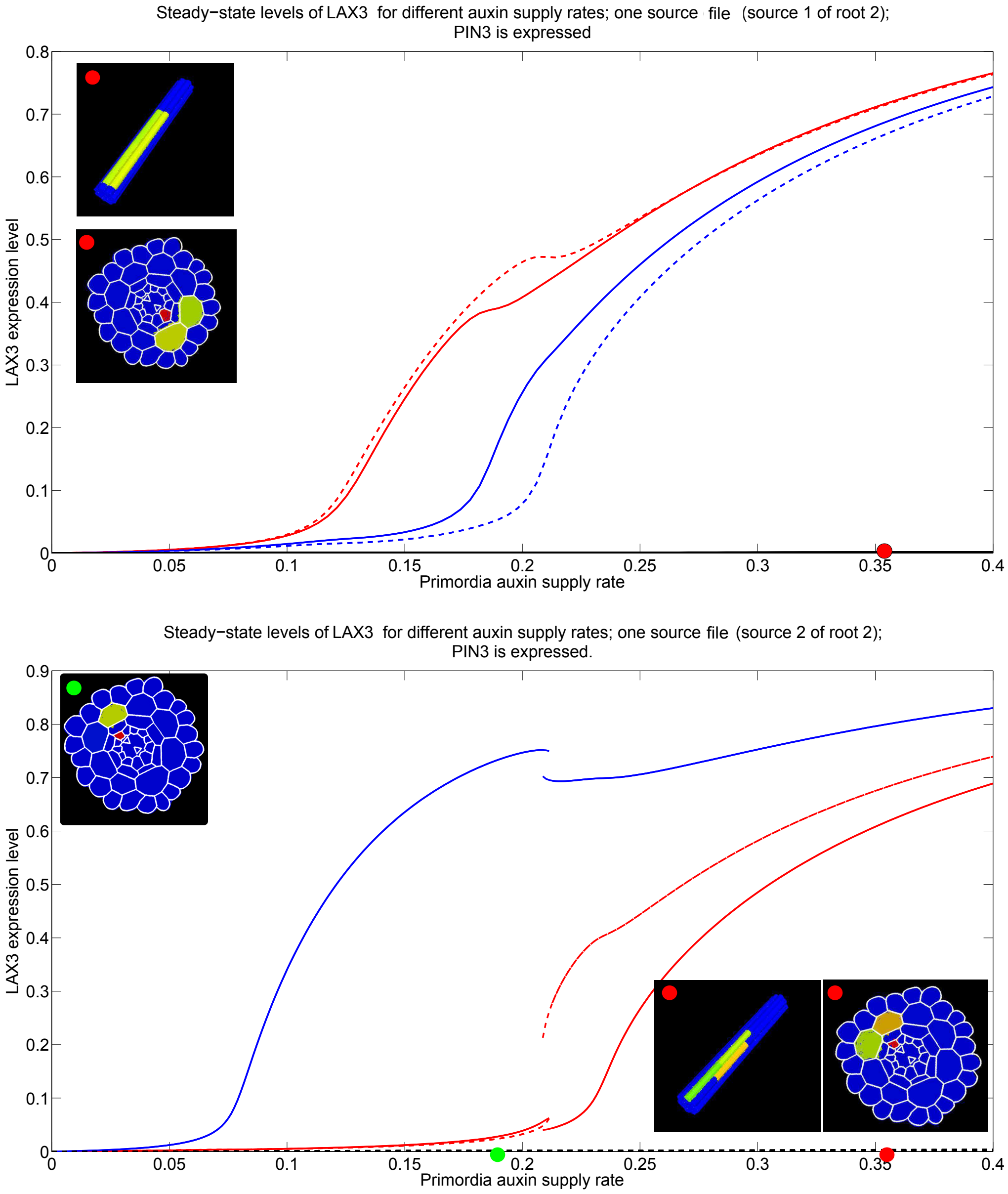

Figure M15: Simulations for root 2 with one XPP cell file acting as a source of auxin and PIN3 is expressed. Colours denote cell file; dashed lines are used to distinguish different cells from the same file. Discontinuities indicate the existence of a bifurcation. Coloured dots indicate level of auxin supply used for the model solution depicted.

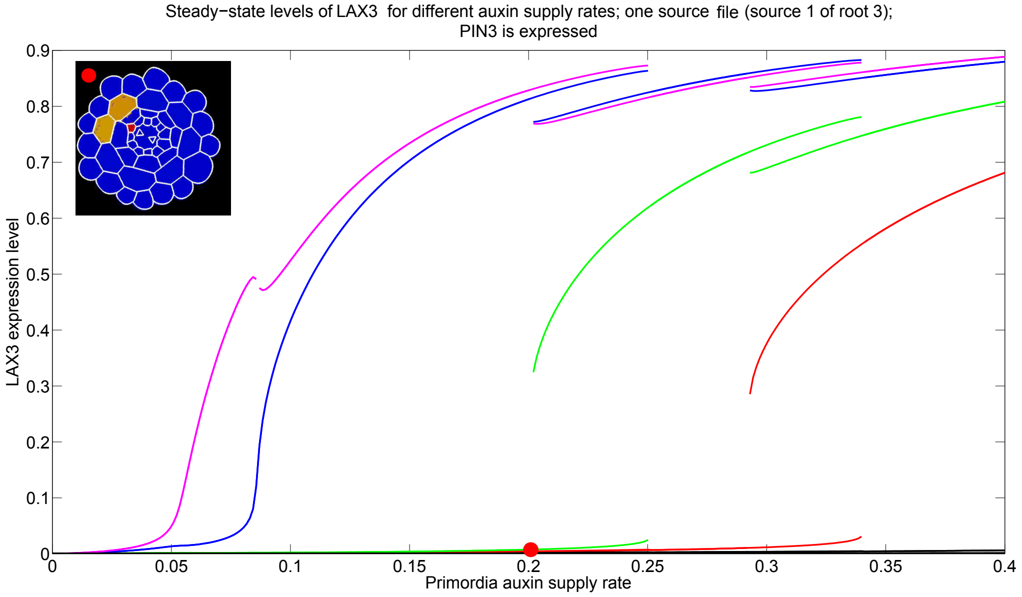

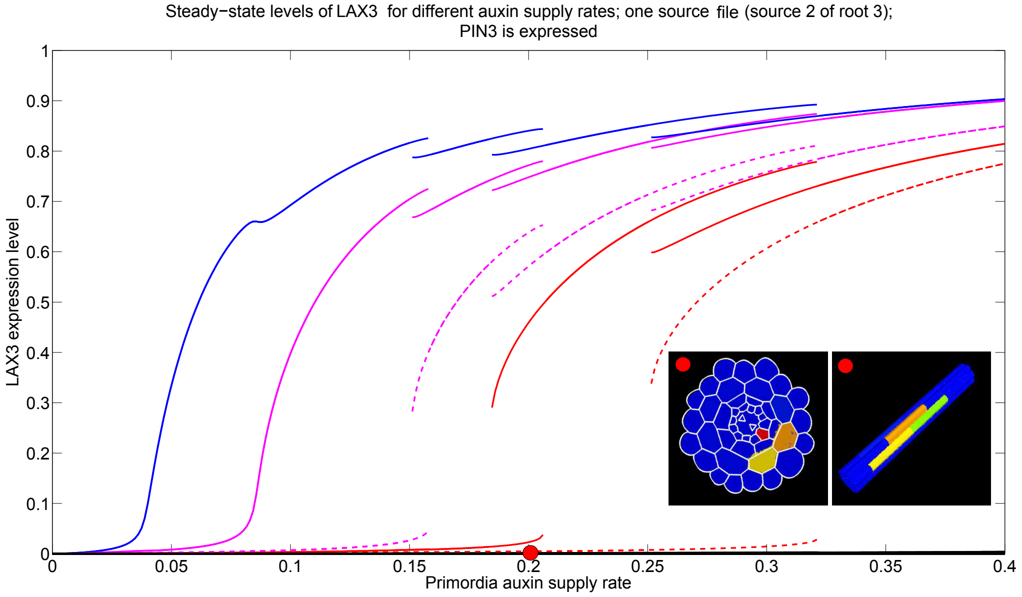

Figure M16: Simulations for root 3 with one XPP cell file acting as a source of auxin and PIN3 is expressed. Colours denote cell file; dashed lines are used to distinguish different cells from the same file. Discontinuities indicate the existence of a bifurcation. Coloured dots indicate level of auxin supply used for the model solution depicted.

7.5.2 Three source files

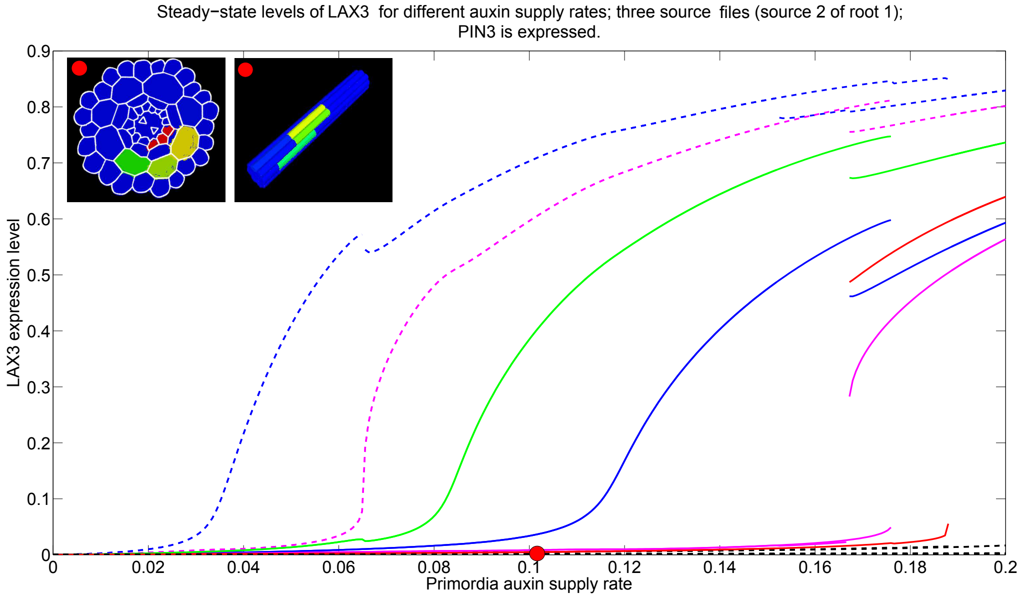

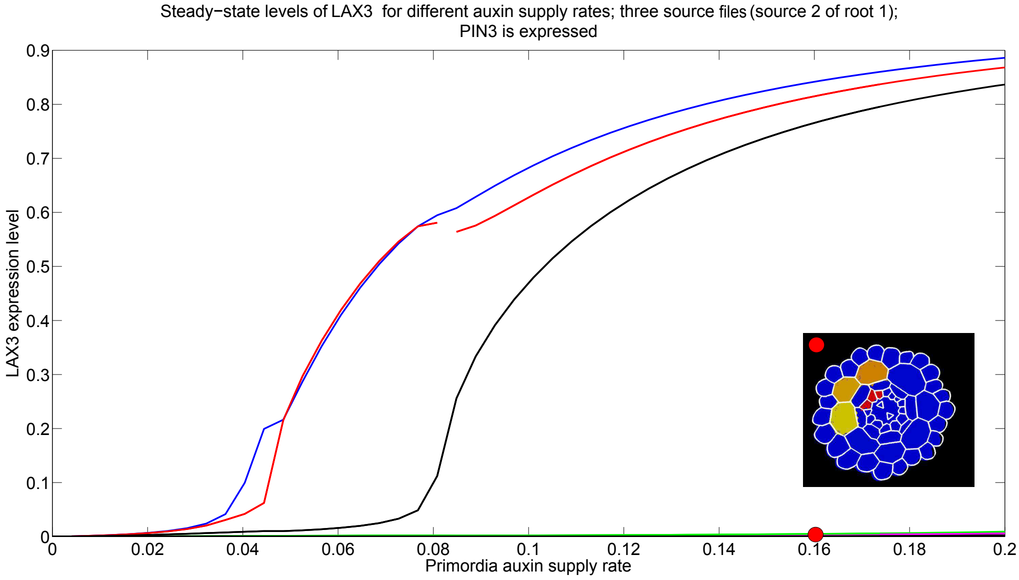

Figure M17: Simulations for root 1 with three XPP cell files acting as a source of auxin and PIN3 is expressed. Colours denote cell file; dashed lines are used to distinguish different cells from the same file. Discontinuities indicate the existence of a bifurcation. Coloured dots indicate level of auxin supply used for the model solution depicted.

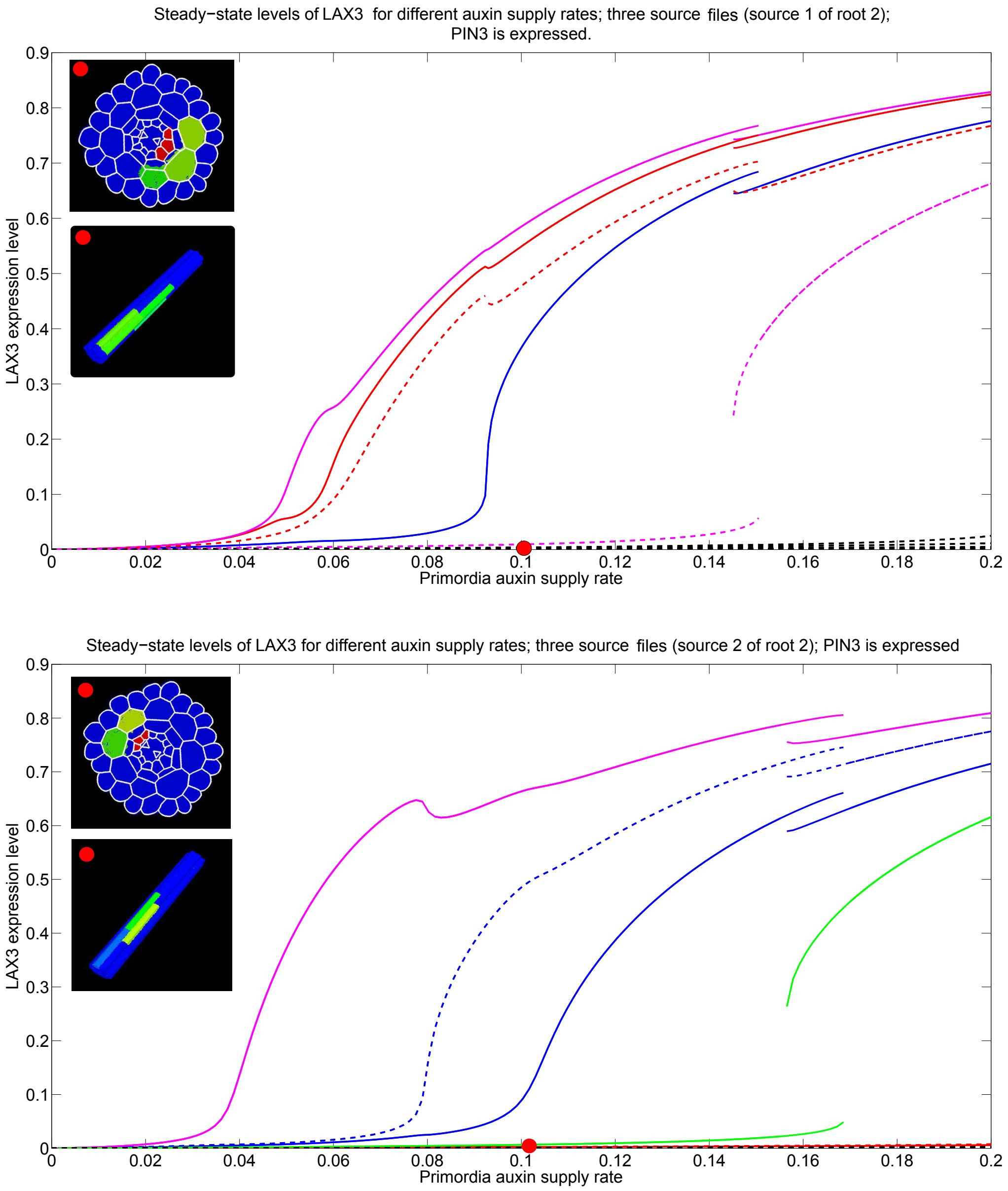

Figure M18: Simulations for root 2 with three XPP cell files acting as a source of auxin and PIN3 is expressed. Colours denote cell file; dashed lines are used to distinguish different cells from the same file. Discontinuities indicate the existence of a bifurcation. Coloured dots indicate level of auxin supply used for the model solution depicted.

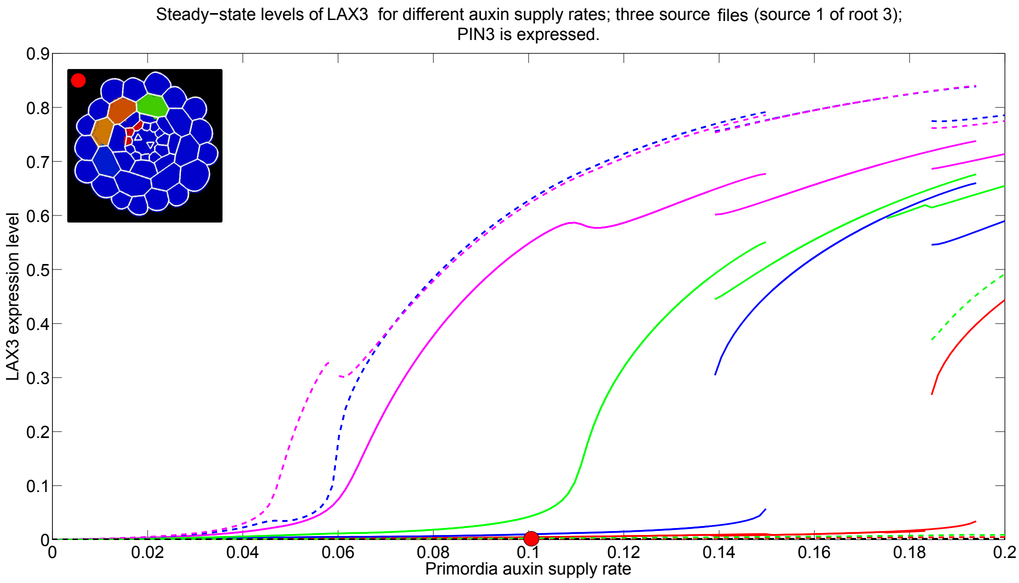

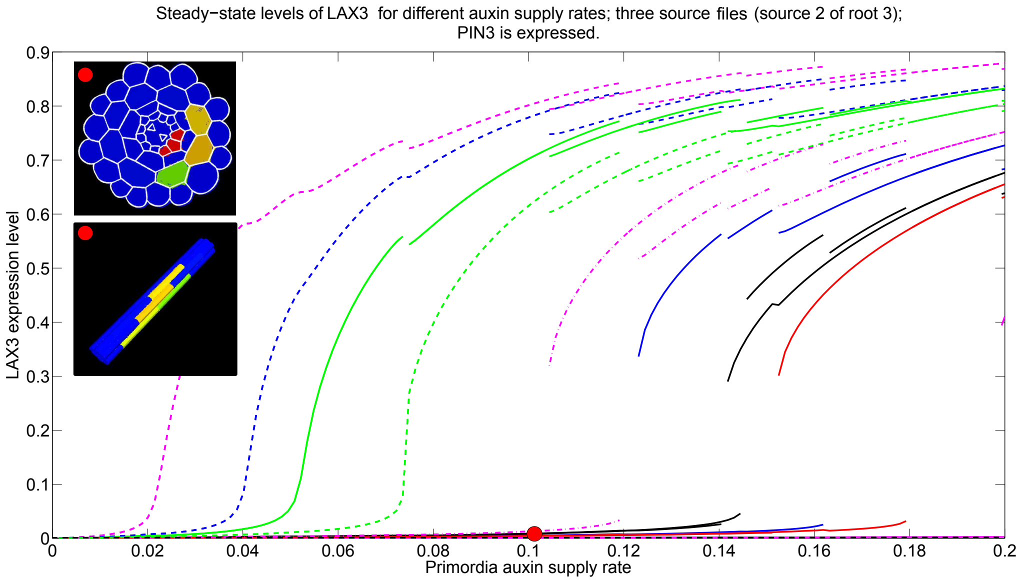

Figure M19: Simulations for root 3 with three XPP cell files acting as a source of auxin and PIN3 is expressed. Colours denote cell file; dashed lines are used to distinguish different cells from the same file. Discontinuities indicate the existence of a bifurcation. Coloured dots indicate level of auxin supply used for the model solution depicted.

7.6 Simulatons for model version three (LAX3 and PIN3 function blocked)

7.6.1 One source file

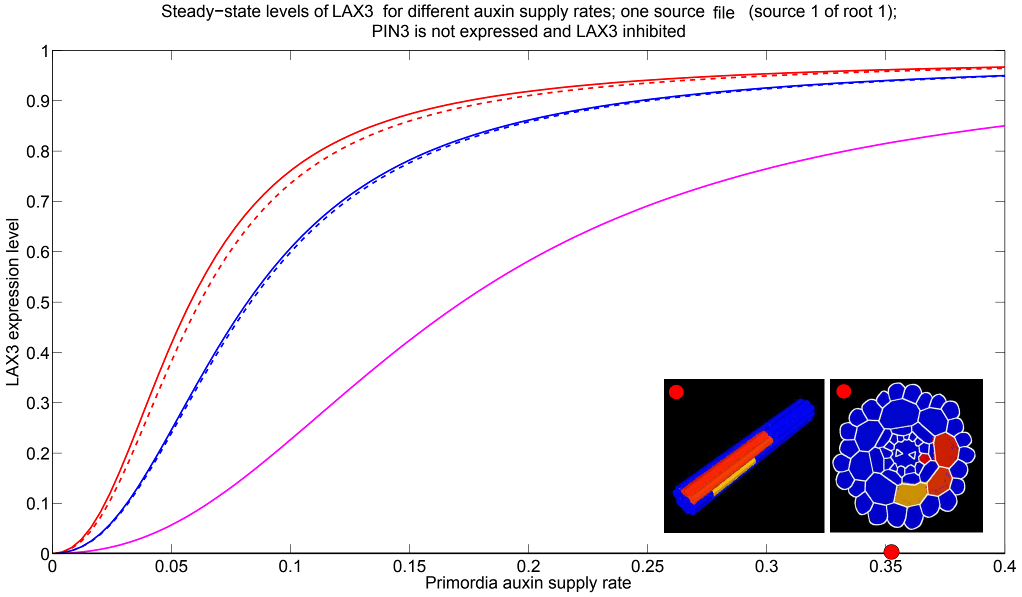

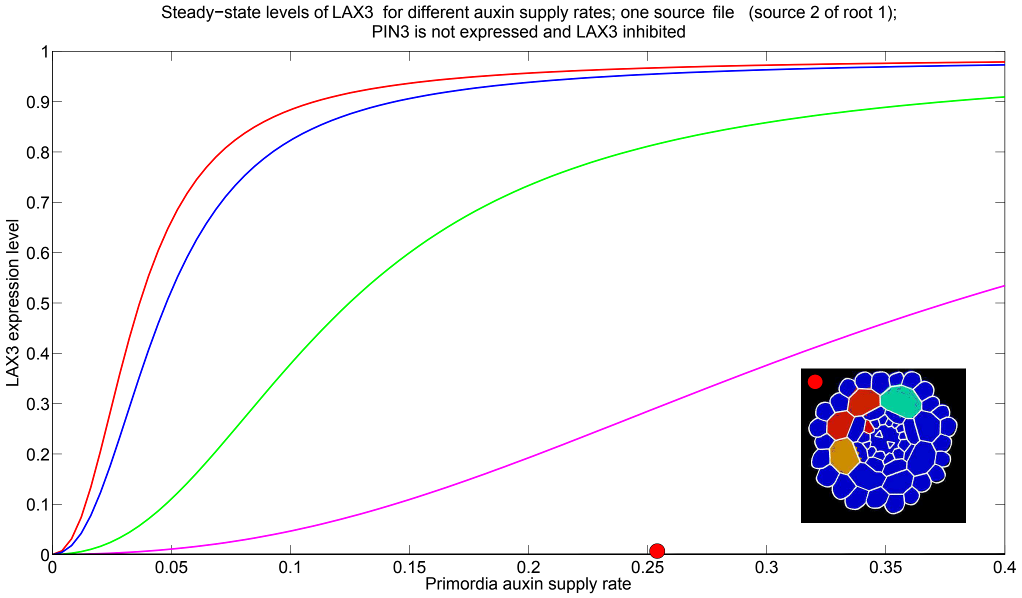

Figure M20: Simulations for root 1 with one XPP cell file acting as a source of auxin and PIN3 is expressed. Colours denote cell file; dashed lines are used to distinguish different cells from the same file. Discontinuities indicate the existence of a bifurcation. Coloured dots indicate level of auxin supply used for the model solution depicted.

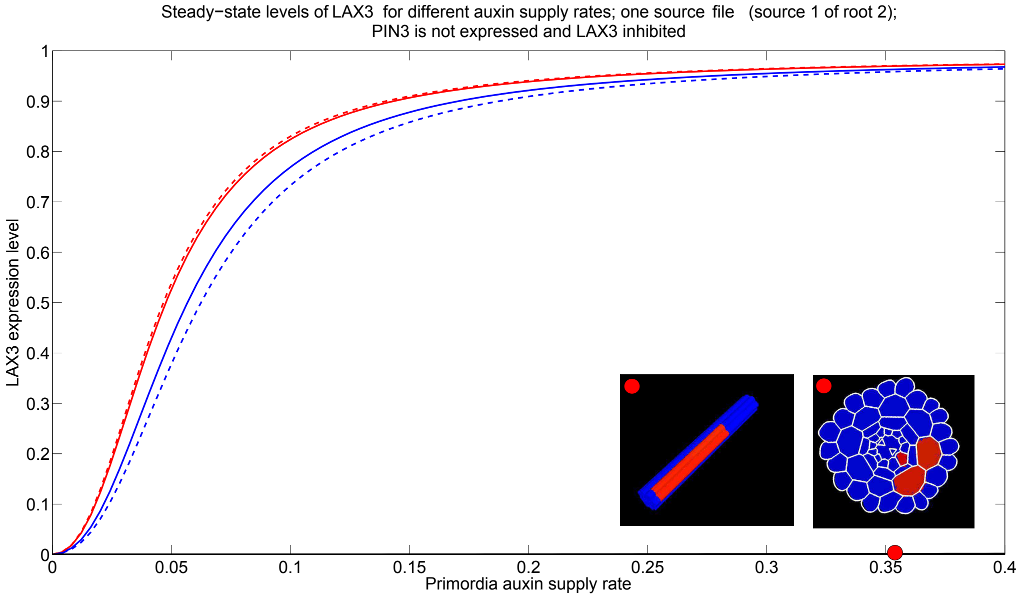

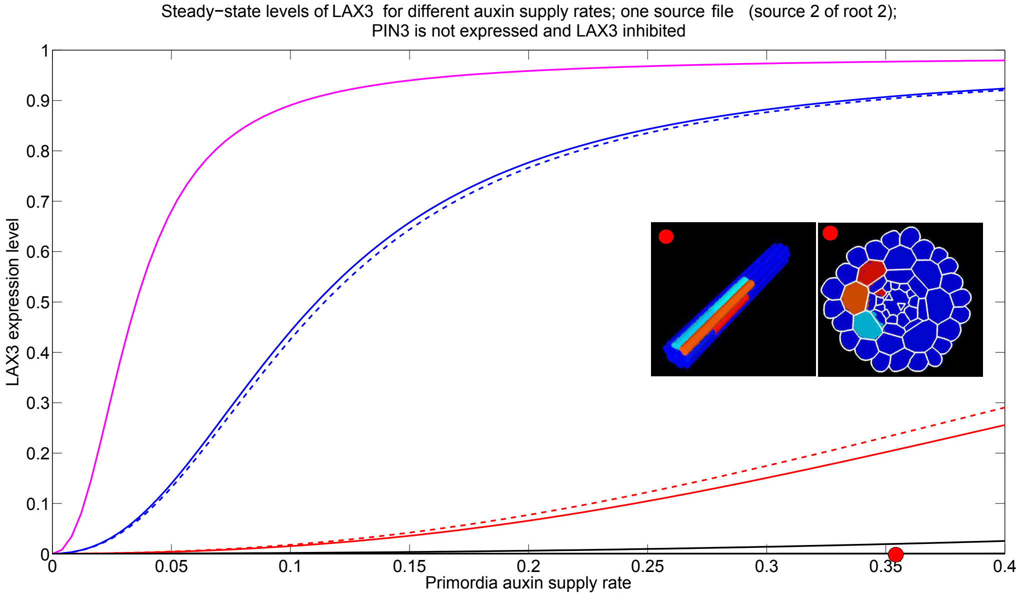

Figure M21: Simulations for root 2 with one XPP cell file acting as a source of auxin and PIN3 is expressed. Colours denote cell file; dashed lines are used to distinguish different cells from the same file. Discontinuities indicate the existence of a bifurcation. Coloured dots indicate level of auxin supply used for the model solution depicted.

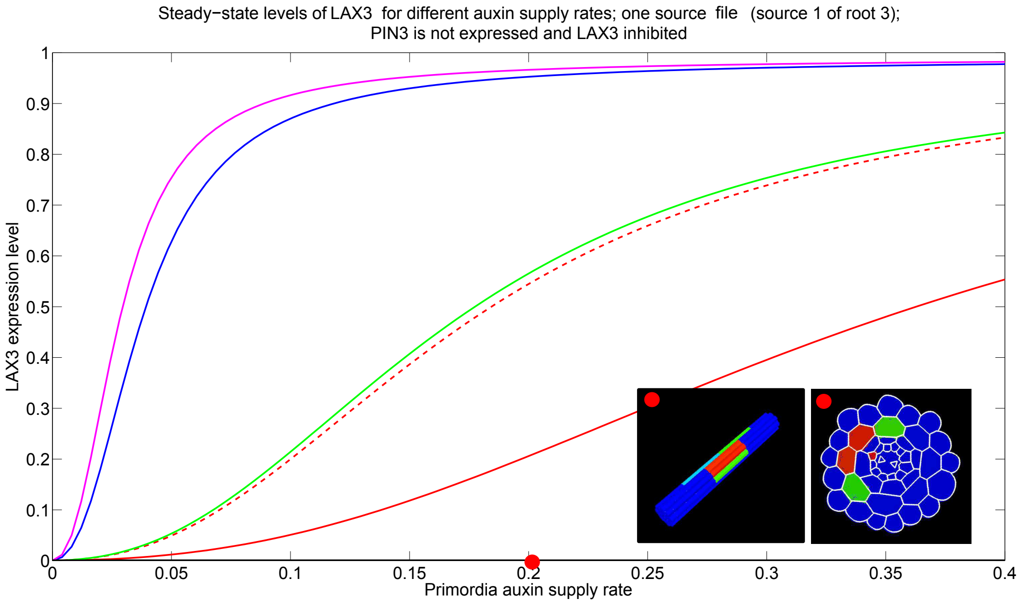

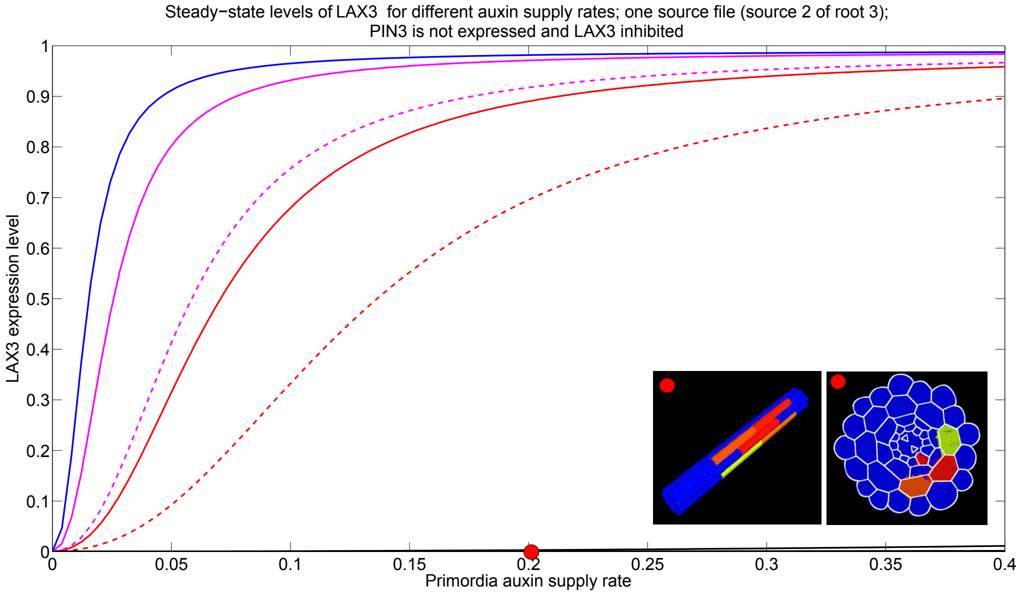

Figure M22: Simulations for root 3 with one XPP cell file acting as a source of auxin and PIN3 is expressed. Colours denote cell file; dashed lines are used to distinguish different cells from the same file. Discontinuities indicate the existence of a bifurcation. Coloured dots indicate level of auxin supply used for the model solution depicted.

7.6.2 Three source files

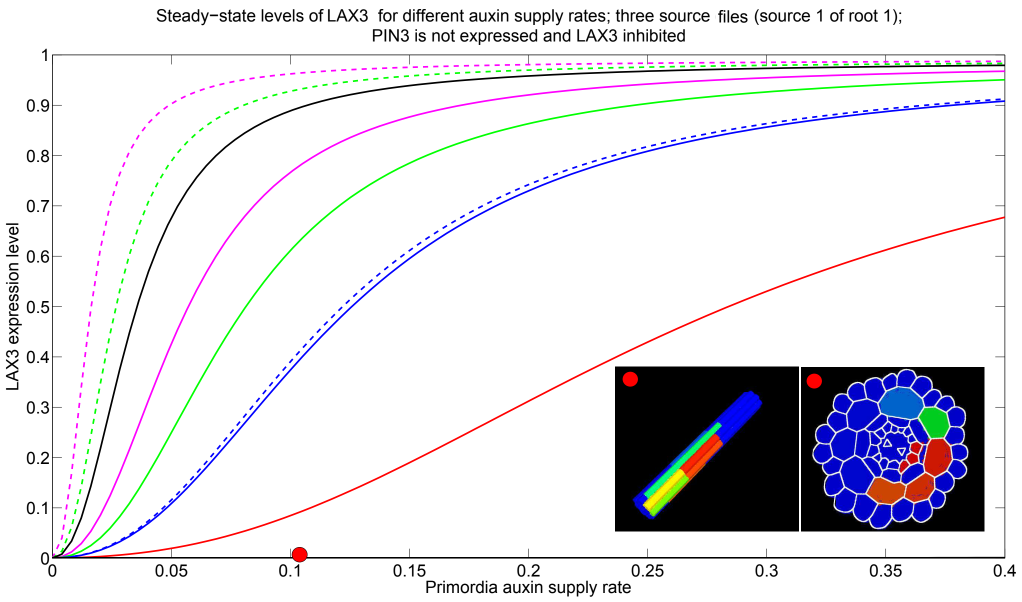

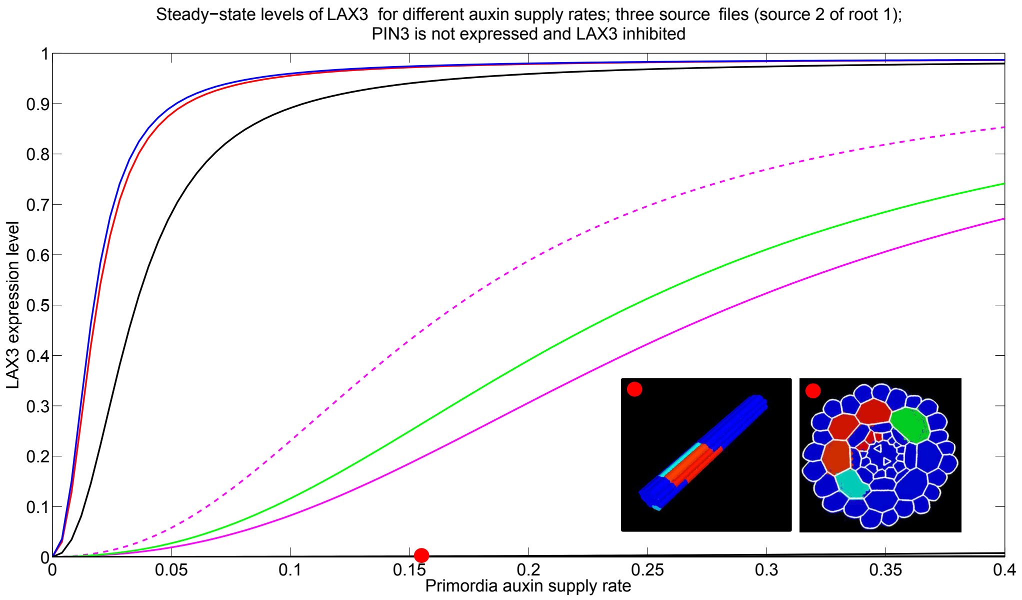

Figure M23: Simulations for root 1 with three XPP cell files acting as a source of auxin and PIN3 is expressed. Colours denote cell file; dashed lines are used to distinguish different cells from the same file. Discontinuities indicate the existence of a bifurcation. Coloured dots indicate level of auxin supply used for the model solution depicted.

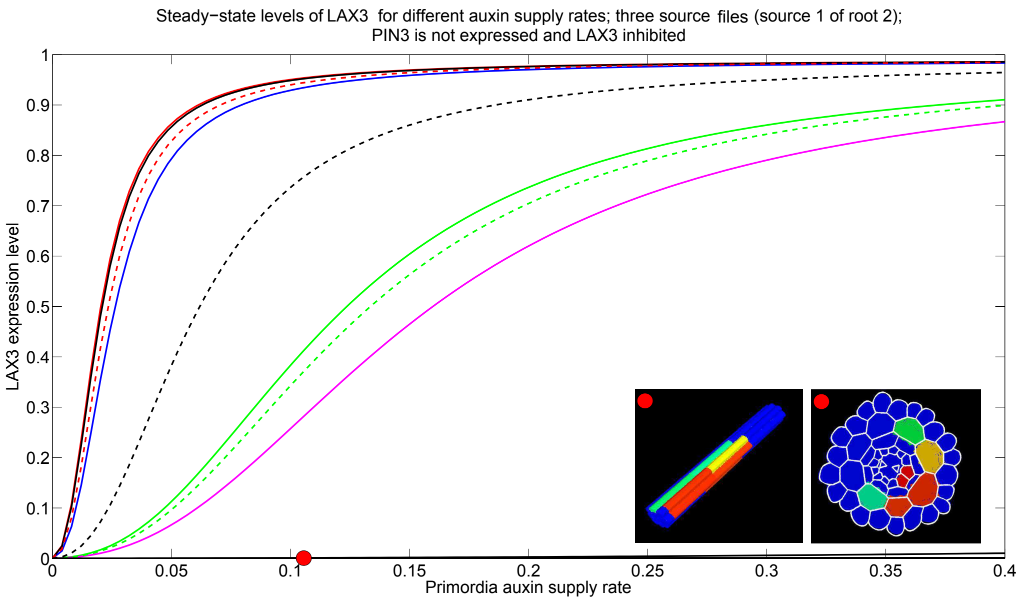

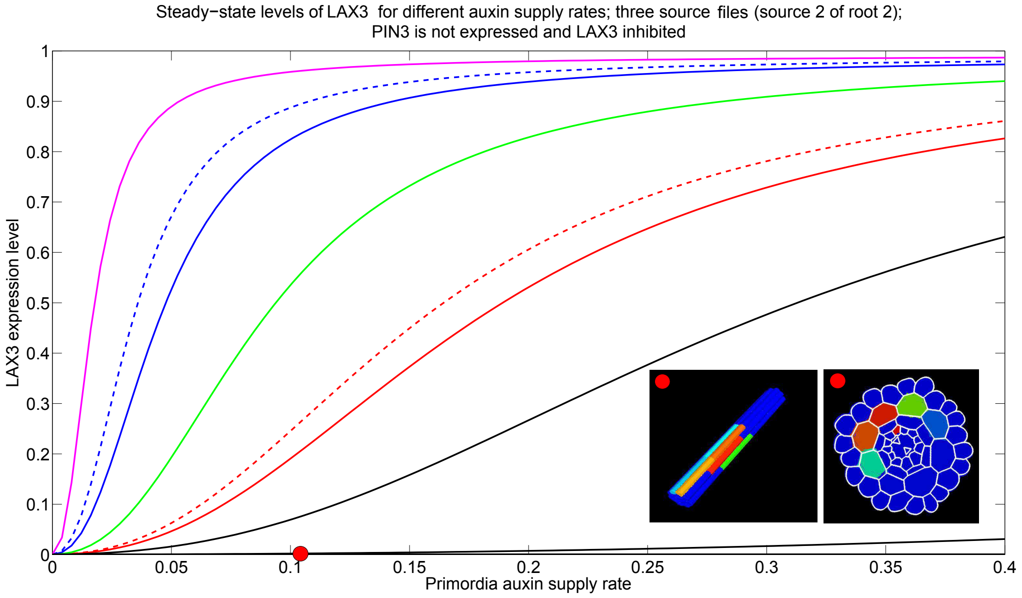

Figure M24: Simulations for root 2 with three XPP cell files acting as a source of auxin and PIN3 is expressed. Colours denote cell file; dashed lines are used to distinguish different cells from the same file. Discontinuities indicate the existence of a bifurcation. Coloured dots indicate level of auxin supply used for the model solution depicted.

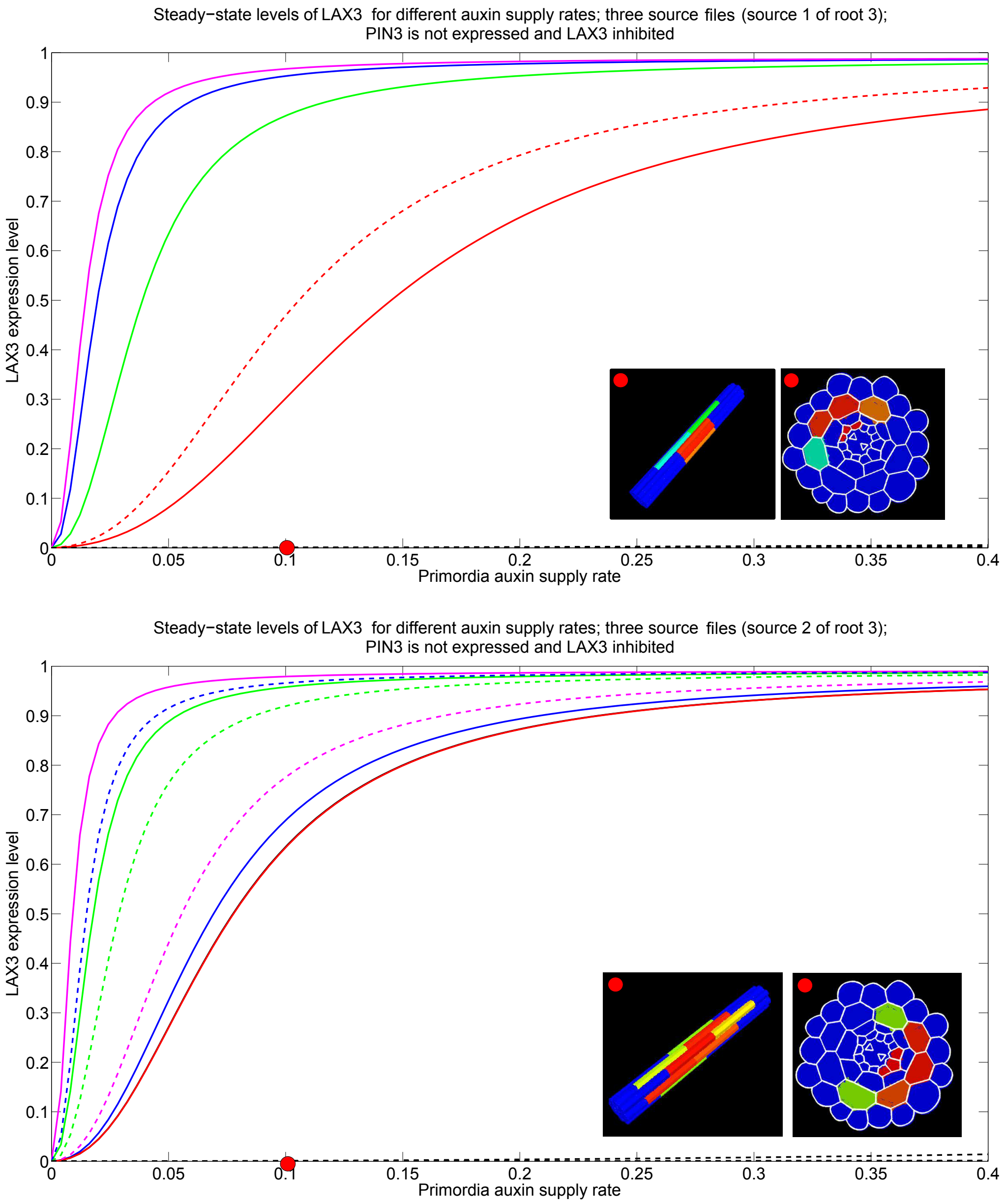

Figure M25: Simulations for root 3 with three XPP cell files acting as a source of auxin and PIN3 is expressed. Colours denote cell file; dashed lines are used to distinguish different cells from the same file. Discontinuities indicate the existence of a bifurcation. Coloured dots indicate level of auxin supply used for the model solution depicted.

References

[1] S. Abel and A. Theologis. Early genes and auxin action. *Plant Physiol.*, 111:9–17, 1996.

[2] P. Alliez, J.D. Biissonnat, and M. Yvinec. Cgal-mesh an open platform for mesh generation.

[3] L.R. Band and J.R. King. Multiscale modelling of auxin transport in the plant-root elongation zone. *Journal of Mathematical Biology*, pages 1–43, 2011.

[4] Eva Benkova, Marta Michniewicz, Michael Sauer, Thomas Teichmann, Daniela Seifertova, Gerd Jurgens, and Jiri Friml. Local, efflux-dependent auxin gradients as a common module for plant organ formation. *Cell*, 115(5):591–602, 2003.

[5] T. Berleth, E. Scarpella, and P. Prusinkiewicz. Towards the systems biology of auxin-transport-mediated patterning. *Trends Plant Sci.*, 12:151–159, 2007.

[6] S. Dharmasisi and M. Estelle. The role of regulated protein degradation in auxin response. *Plant Mol Biol.*, 49:401–409.

[7] J.M. Fasano, S.J. Swanson, E.B. Blancaflor, P.E. Dowd, T. Kao, and S. Gilroy. Changes in root cap ph are required for the gravity response of the arabidopsis root. *The Plant Cell Online*, 13(4):907–922, 2001.

[8] P.J. Frey. Medit: An interactive mesh visualization software. 2001.

[9] M. H. M. Goldsmith, T. H. Goldsmith, and M. H. Martin. Mathematical analysis of the chemosmotic polar diffusion of auxin through plant tissues. *P. Natl Acad. Sci. USA*, 78:976–980, 1981.

[10] V.A. Grieneisen, J. Xu, A.F.M. Marée, P. Hogeweg, and B. Scheres. Auxin transport is sufficient to generate a maximum and gradient guiding root growth. *Nature*, 449(7165):1008–1013, 2007.

[11] T. Guilfoyle, G. Hagen, T. Ulmasov, and J. Murfett. How does auxin turn on genes? *Plant Physiol.*, 118:341–347, 1998.

[12] G. Hagen and T. Guilfoyle. Auxin-responsive gene expression: genes, promoters and regulatory factors. *Plant Mol Biol.*, 49:373–385, 2002.

[13] H. Jönsson, M.G. Heisler, B.E. Shapiro, E.M. Meyerowitz, and E. Mjolsness. An auxin-driven polarized transport model for phyllotaxis. *Proceedings of the National Academy of Sciences of the United States of America*, 103(5):1633, 2006.

[14] H. Jönsson and P. Krupinski. Modeling plant growth and pattern formation. *Curr. Opin. Plant Biol.*, 13:5–11, 2010.

[15] J. Kleine-Vehn and J. Friml. Polar targeting and endocytic recycling in auxin-dependent plant development. *Annual review of cell and developmental biology*, 24:447–473, 2008.

[16] E. M. Kramer. Computer models of auxin transport: a review and commentary. *J. Exp. Bot.*, 59:45–53, 2008.

[17] E.M. Kramer. Pin and aux/lax proteins: their role in auxin accumulation. *Trends in plant science*, 9(12):578–582, 2004.

[18] E.M. Kramer and M.J. Bennett. Auxin transport: a field in flux. *Trends in plant science*, 11(8):382–386, 2006.

[19] M. Laskowski, V.A. Grieneisen, H. Hofhuis, A. Colette, P. Hogeweg, A.F.M. Marée, and B. Scheres. Root system architecture from coupling cell shape to auxin transport. *PLoS Biology*, 6(12):e307, 2008.

[20] M.T. Loper and R.M. Spanswick. Auxin transport in suspension-cultured soybean root cells: I. characterization. *Plant physiology*, 96(1):184, 1991.

[21] A. Marchant, R. Bhalerao, I. Casimiro, J. Eklöf, P.J. Casero, M. Bennett, and G. Sandberg. Aux1 promotes lateral root formation by facilitating indole-3-acetic acid distribution between sink and source tissues in the arabidopsis seedling. *The Plant Cell Online*, 14(3):589, 2002.

[22] AM Middleton, JR King, MJ Bennett, and MR Owen. Mathematical modelling of the aux/iaa negative feedback loop. *Bulletin of mathematical biology*, 72(6):1383–1407, 2010.

[23] F. Perrine-Walker, P. Doumas, M. Lucas, V. Vaissayre, N.J. Beauchemin, L.R. Band, J. Chopard, A. Crabos, G. Conejero, B. Péret, et al. Auxin carriers localization drives auxin accumulation in plant cells infected by frankia in casuarina glauca actinorhizal nodules. *Plant physiology*, 154(3):1372–1380, 2010.

[24] R. S. Smith and E. M. Bayer. Auxin transport-feedback models of patterning in plants. *Plant Cell Environ.*, 32:1258–1271, 2009.

[25] S. Stoma, M. Lucas, J. Chopard, M. Schaedel, J. Traas, and C. Godin. Flux-based transport enhancement as a plausible unifying mechanism for auxin transport in meristem development. *PLoS Comput. Biol.*, 4:e1000207, 2008.

[26] K. Swarup, E. Benková, R. Swarup, I. Casimiro, B. Péret, Y. Yang, G. Parry, E. Nielsen, I. De Smet, S. Vanneste, et al. The auxin influx carrier lax3 promotes lateral root emergence. *Nature Cell Biology*, 10(8):946–954, 2008.

[27] R. Swarup, E.M. Kramer, P. Perry, K. Knox, H.M.O. Leyser, J. Haseloff, G.T.S. Beemster, R. Bhalerao, and M.J. Bennett. Root gravitropism requires lateral root cap and epidermal cells for transport and response to a mobile auxin signal. *Nature Cell Biology*, 7(11):1057, 2005.

[28] W.D. Teale, I.A. Paponov, and Palme K. Auxin in action: signalling, transport and the control of plant growth and development. *Nat Rev Mol Cell Biol.*, 7:847–859, 2006.

[29] T. Ulmasov, G. Hagen, and T.J. Guilfoyle. Dimerization and DNA binding of auxin response factors. *Plant J.*, 19:309–319, 1999.

Sup Figure S1

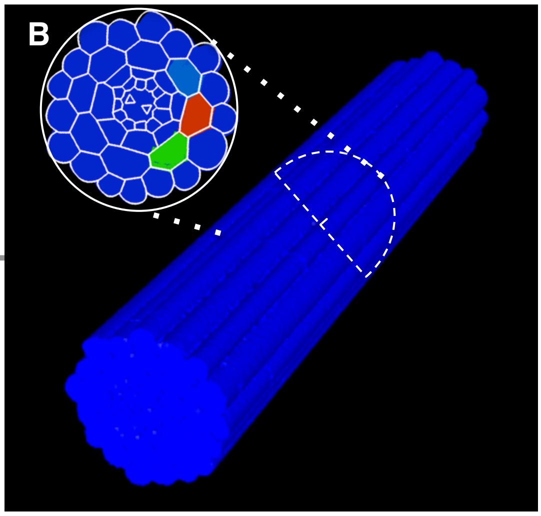

LAX3 expression levels predicted by model version one. Cross section position as indicated (see also Figure 1).

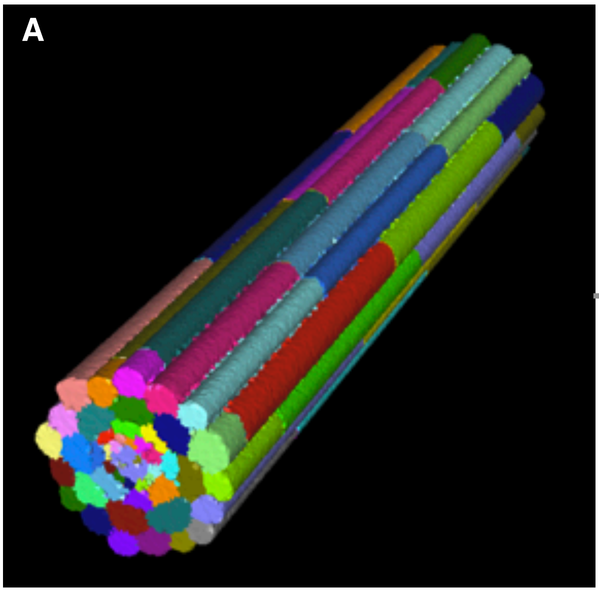

Original 3D mesh

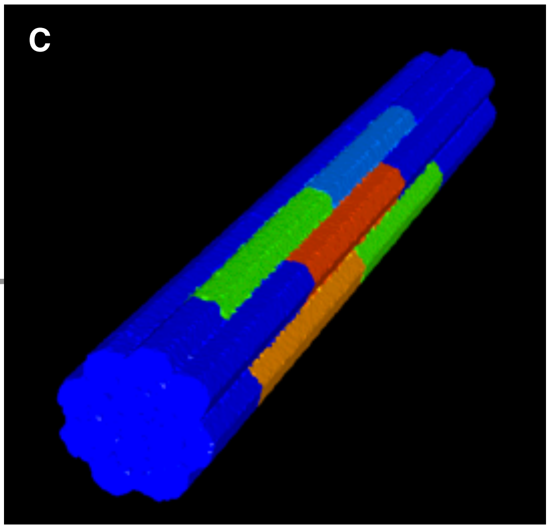

LAX3 expression levels predicted by model version one (as above - now with epidermis removed for visual clarity).

Supplementary Figure S1 (A) Original 3D mesh generated using the scheme illustrated in Figure 4. **(B)** Cell faces are coloured according to the predicted LAX3 expression pattern. Since LAX3 is not expressed in the epidermis, this blocks a direct view of the cortical expression. We therefore generate cross sections showing the circumferential distribution of LAX3. **(C)** For greater clarity, when the pattern spans more than one cell per file, we also remove the epidermis. For the case presented here, we see the LAX3 expression can extend both longitudinally (more than one cell per file) as well as circumferentially (to many files), thus generating a 3D expression pattern – as is observed experimentally (see Figure 1).

Sup Figure S2

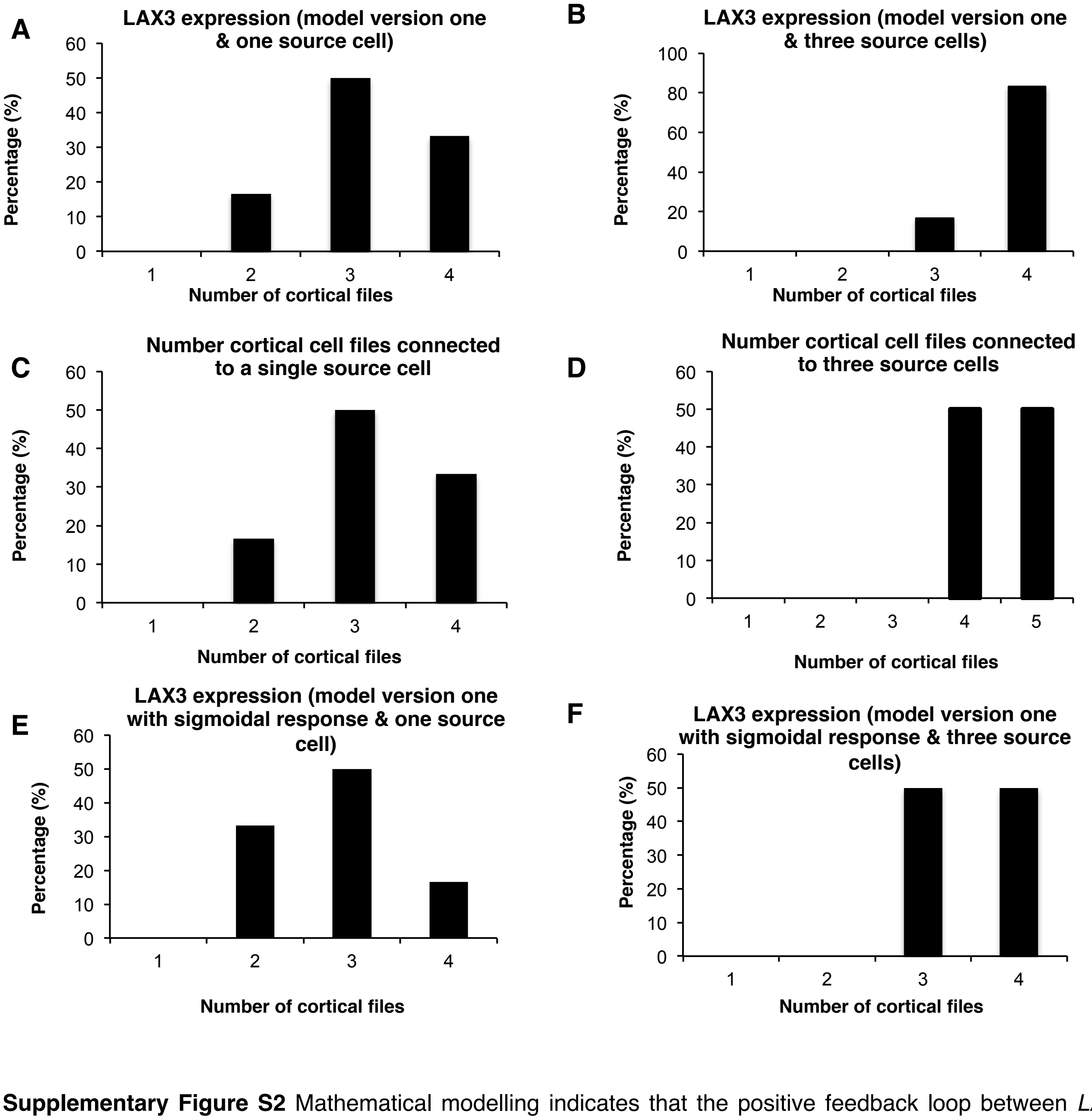

Supplementary Figure S2 Mathematical modelling indicates that the positive feedback loop between *LAX3* expression levels and auxin accumulation cannot account for the restriction of LAX3 to just two cell files. **(A)-(B)** Total number of cortical cell files predicted by model version one to express LAX3 (at steady-state), assuming the auxin is provided by one (A) or three (B) XPP cell files act as an auxin source. **(C)-(D)** Number of cortical cell files that connect (via the endodermis) to either a single source cell (C) or three source cells (D). **(E)-(F)** As (A)-(B), but instead when LAX3 is assumed to give a sigmoidal response to increasing levels of auxin (see Figure 4H and main text).

Sup Figure S3

A

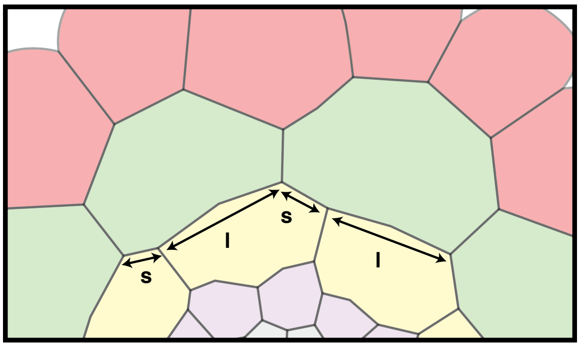

s : short endodermal/cortical cell contact

l : long endodermal/cortical cell contact

B

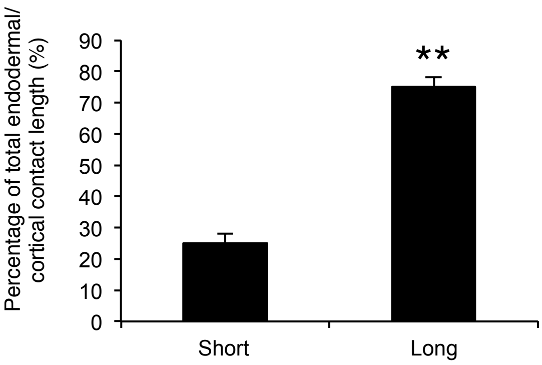

Supplementary Figure S3 A typical endodermal cell shares contact with two cortical cells. (A) Manually segmented image of a root cross section showing the short (s) and long (l) contacts between endodermal and cortical cells. (**B**) Percentage of total contact length is conserved across several root sections (n=22) demonstrating the existence of a conserved pattern. Root cross sections were analysed with the ImageJ image software and short to long ratios were plotted as average values (± sem). Asterisks indicate a significant difference with corresponding control experiment by Student's t-test (**: $p < 0.01$; n=44).

Sup Figure S4

A

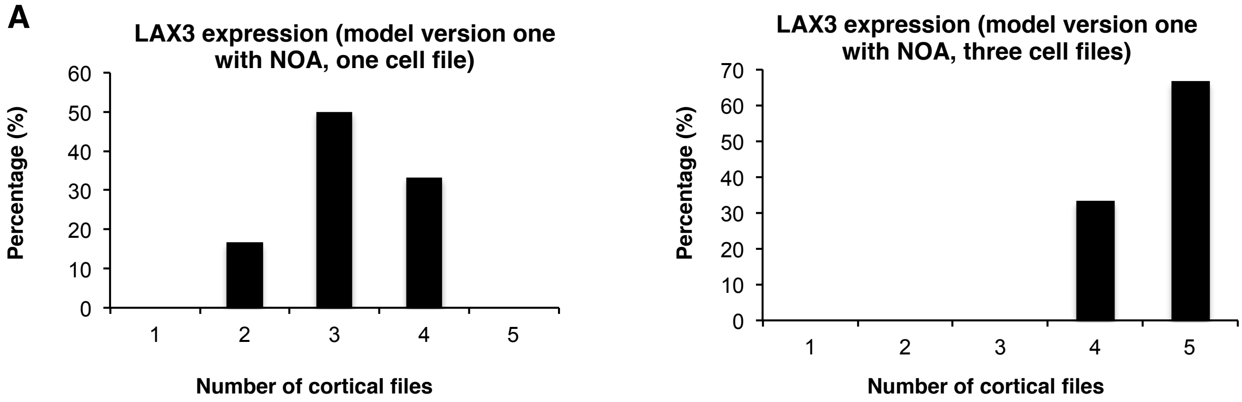

B

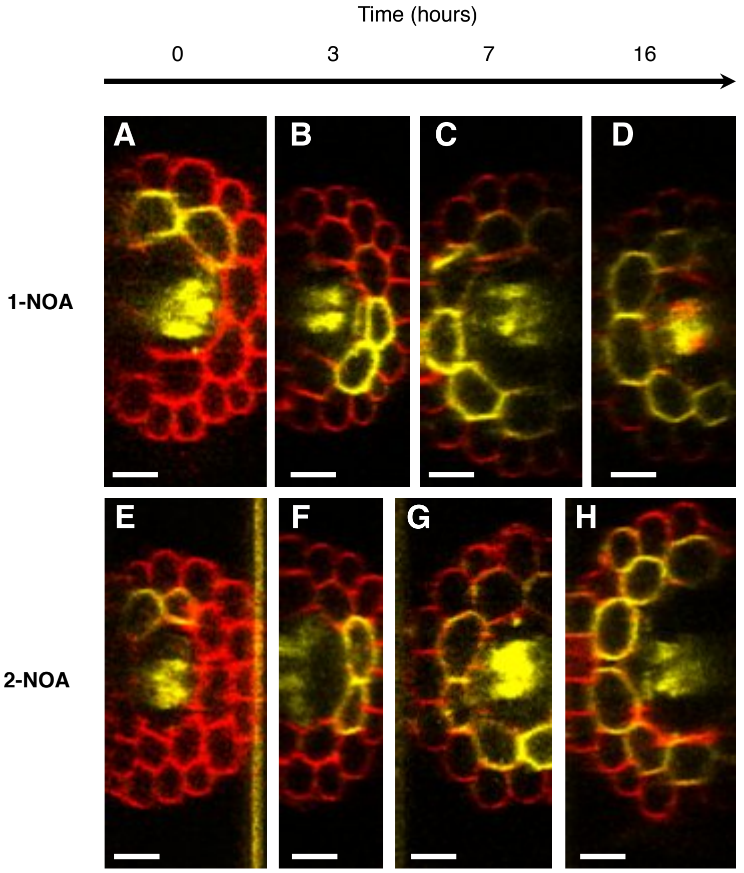

Supplementary Figure S4 Blocking auxin influx activity triggers a spread of *LAX3* expression. (**A**) Total number of cell files predicted by model version one to express LAX3 (at steady-state) when auxin influx transporters (including LAX3) are blocked chemically with NOA. Shown for when either one or three XPP cell files act as an auxin source. (**B**) Treating plants with 1-NOA or 2-NOA resulted in a spread of LAX3-YFP accumulation with similar dynamics. 5 day-old plants were transferred on medium supplemented with 10μM of 1-NOA or 2-NOA and imaged by laser scanning confocal microscopy at 0, 3, 7 and 16 hours. Bars are 25 μm.

Sup Figure S5

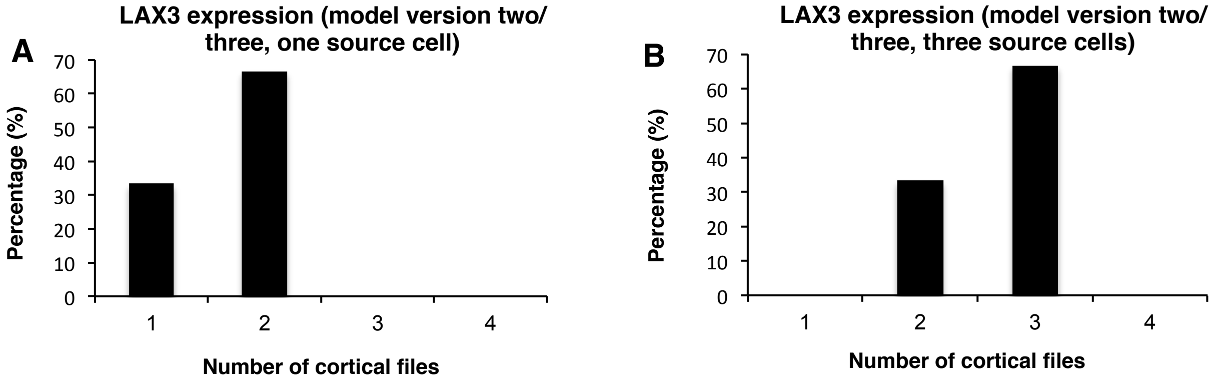

Supplementary Figure S5 (**A**-**B**) Total number of cell files predicted to express LAX3 (at steady-state) when either one (A) or three (B) XPP cell files act as source of auxin. These are valid for both model versions two and three since the steady-state behaviour of both models is the same.

Sup Figure S6

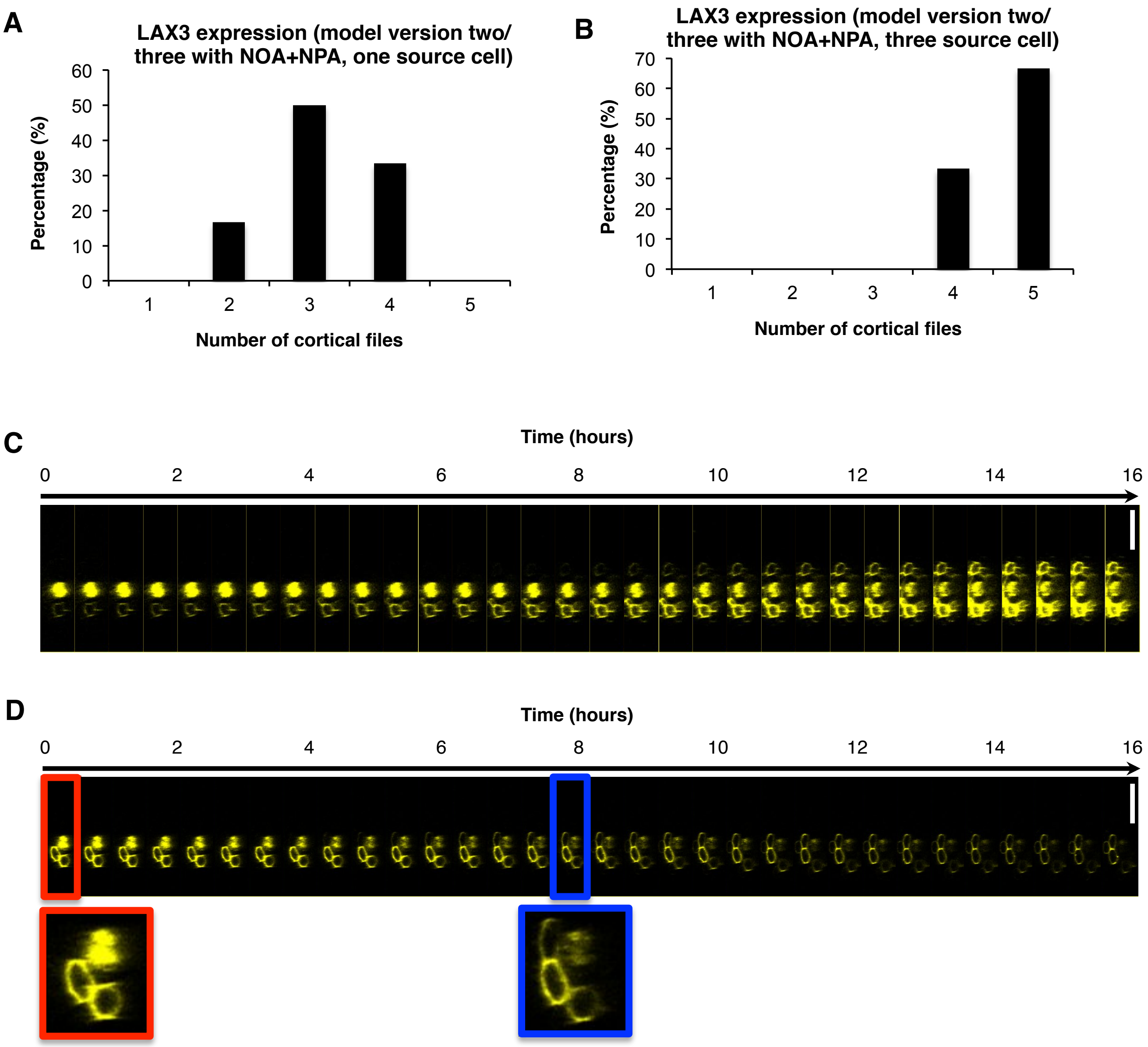

Supplementary Figure S6 Differences in the spatial expression of LAX3-YFP following combined 2-NOA and NPA treatments. (**A**-**B**) Total number of cell files predicted to express LAX3 (at steady-state) when auxin influx and efflux carriers are blocked chemically with NOA and NPA when either one (A) or three (B) XPP cell files act as source of auxin. Note that these are valid for both model versions two and three since the steady-state behaviour of both models is the same. (**C**) Time course experiment showing the spread of LAX3-YFP upon 10μM 2-NOA treatment starting at *ca.* 4 hours and originating from the two xylem poles. (**D**) Time course experiment showing the spread of LAX3-YFP upon 10μM 2-NOA and 10μM NPA treatment starting at *ca.* 6 hours. Blow up of images shows spread of LAX3 expression from two cell files (red) to three (blue) within the first few hours of treatment. Bars are 75 μm.

Sup Figure S7

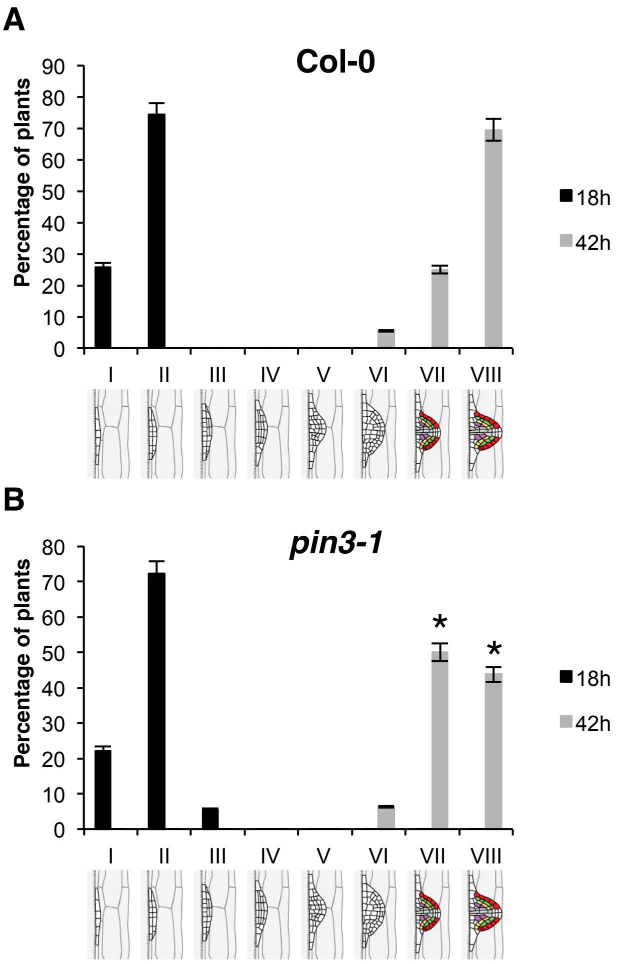

Supplementary Figure S7 Loss-of-function *pin3-1* mutants display delayed LR emergence. (**A-B**) LR formation was induced and synchronised by giving the plants a 90° gravistimulus 3 days after germination. LRP were scored into developmental stages 18 and 42 hours post-gravistimulation (hpg). Wild-type (Col-0) plants show accumulation of stage I and II LRP at 18 hpg and accumulation of mainly stage VIII LRP at 42 hpg (A). Loss-of-function *pin3-1* mutants display accumulation of stage I and II LRP at 18 hpg and accumulation of stage VII and VIII LRP at 42 hpg (B). Asterisks indicate a significant difference with corresponding control experiment by Student's t-test (*: $p < 0.05$; n=20).

Sup Figure S8

**Gene name** (AGI)	**Forward primer**	**Reverse primer**
LAX3 (At1g77690)	5'-tcaccattgcttcactccttc-3'	5'-aagcaccattgtggttggac-3'
PG (At5g14650)	5'-catcgatggacgaggatca-3'	5'-cctcaaagctgttggtttgg-3'
PIN3 (At1g70940)	5'-cccagatcaatctcacaacg-3'	5'-ccggcgaaactaaattgttg-3'

Supplementary Figure S8 Primers used for quantitative RT-PCR.
